# Supplementary material for: Non-invasive in vivo sensing of bacterial implant infection using catalytically-optimised gold nanocluster-loaded liposomes for urinary readout
Source: Nat Commun. 2024 Nov 28;15:10321. doi: 10.1038/s41467-024-53537-2 (PMC11605077; doi:10.1038/s41467-024-53537-2)
Supplement: Supplementary file 1 — Supplementary information [file 41467_2024_53537_MOESM1_ESM.pdf]

Supplementary information

Non-invasive *in vivo* sensing of bacterial implant infection using catalytically-optimised gold nanocluster-loaded liposomes for urinary readout

Table of Contents

Supplementary Table 1	S2
Supplementary Figure 1	S3
Supplementary Figure 2	S4
Supplementary Figure 3	S5
Supplementary Figure 4	S6
Supplementary Figure 5	S8
Supplementary Figure 6	S10
Supplementary Figure 7	S11
Supplementary Figure 8	S12
Supplementary Figure 9	S13
Supplementary Figure 10	S14
Supplementary Figure 11	S15
Supplementary Figure 12	S16
Supplementary Figure 13	S17
Supplementary Table 2	S18
Supplementary Figure 14	S19
Supplementary Table 3	S20
Supplementary Figure 15	S21
Supplementary Figure 16	S23
Supplementary Figure 17	S24
Supplementary Figure 18	S25
Supplementary Table 4	S26
Supplementary Figure 19	S27
Supplementary Figure 20	S28
Supplementary Figure 21	S29
Supplementary Figure 22	S30
Supplementary Figure 23	S31
Supplementary Figure 24	S33
Supplementary Figure 25	S34
Supplementary Movie 1	S36
References	S37

Supplementary Table 1. Synthesis conditions for the optimisation of nanocluster catalytic activity (Sodium borohydride was used for strong reducing condition and GSH was used for weak reducing condition).

		Strong reducing condition		Weak reducing condition	
	No.	Samples	Au or Pt : Ligand Ratio	Samples	Au or Pt : Ligand Ratio
Au NCs (H_2O)	1	Au-H_2O-a	1:2	Au-H_2O-a	1:2
	2	Au-H_2O-b	1:1.5	Au-H_2O-b	1:1.5
	3	Au-H_2O-c	1:1	Au-H_2O-c	1:1
Au NCs (EtOH)	4	Au-EtOH-a	1:2	Au-EtOH-a	1:2
	5	Au-EtOH-b	1:1.5	Au-EtOH-b	1:1.5
	6	Au-EtOH-c	1:1	Au-EtOH-c	1:1
Pt NCs (H_2O)	7	Pt-H_2O-a	1:2	Au-H_2O-a	1:2
	8	Pt-H_2O-b	1:1.5	Au-H_2O-b	1:1.5
	9	Pt-H_2O-c	1:1	Au-H_2O-c	1:1
Pt NCs (NaOH)	10	Pt-NaOH-a	1:2	Au-NaOH-a	1:2
	11	Pt-NaOH-b	1:1.5	Au-NaOH-b	1:1.5
	12	Pt-NaOH-c	1:1	Au-NaOH-c	1:1

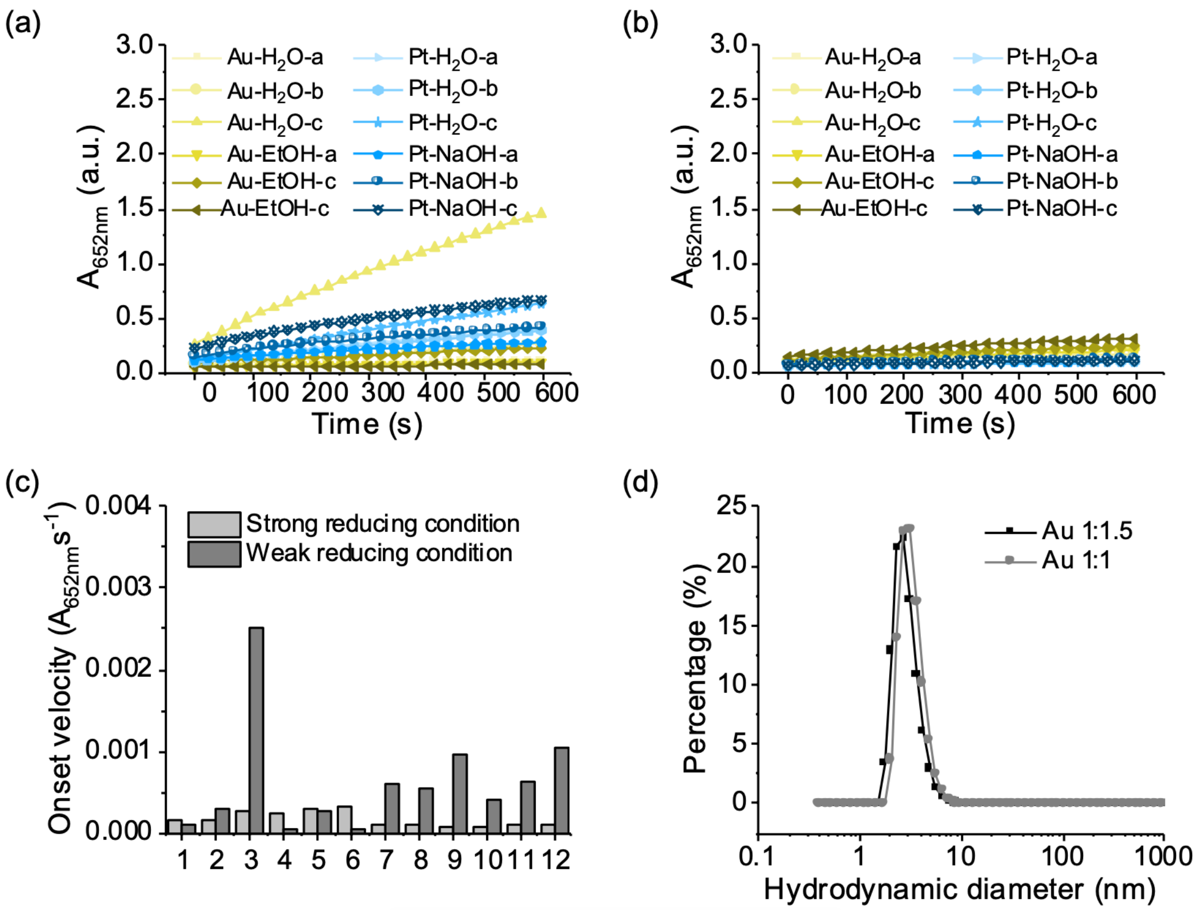

Supplementary Figure 1. Nanocluster synthesis optimisation. (a, b) Catalytic activity (TMB/H_2O_2 system) of gold and platinum nanoclusters synthesised in strong (a) and weak (b) reducing condition, with letters a-c corresponding to different metal-to-GSH ratios (a = 1:2, b = 1:1.5, c = 1:1) and different addition of ethanol or sodium hydroxide (sample numbers 1-12 refer to Supplementary Table 1, curves are mean of n = 2 technical repeats). (c) Onset velocity calculated from TMB assays using AuNCs synthesised under strong and weak reducing condition using linear fits (sample numbers 1-12 refer to Supplementary Table 1, bars are mean of n = 2 technical repeats). (d) Number distribution by dynamic light scattering of AuNCs at gold-to-GSH ratio of 1:1 and 1:1.5 (two optimal nanocluster versions selected from the screening, curves are mean of n = 3 technical repeats).

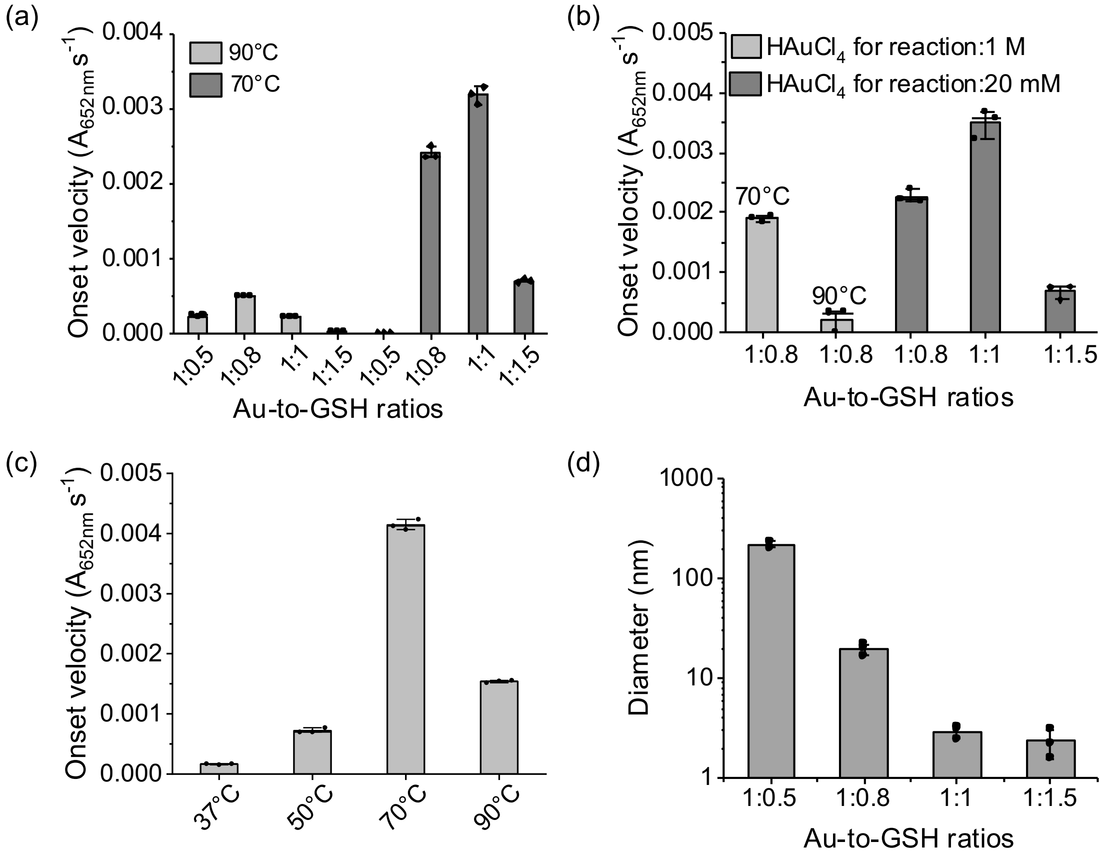

Supplementary Figure 2. Further optimisation of nanocluster synthesis (different gold-to-GSH ratio and temperature under weak reducing condition) and characterisation (TMB and DLS). (a) Catalytic activity of different AuNCs synthesised with various Au-to-GSH ratio at 70 °C and 90 °C. AuNCs exhibited higher catalytic activity when synthesised at 70 °C (mean values ± standard error, n = 3 technical repeats). (b) Comparison of catalytic activity after using different concentrations of initial reaction agents for synthesis of nanoclusters (nanoclusters of 1:0.8 at 70°C and 90 °C were adapted from literature[1] for comparison) (mean values ± standard error, n = 3 technical repeats). (c) The influence of different synthesis reaction temperatures on the catalytic activity of AuNCs. 70 °C was the optimal synthesis temperature (mean values ± standard error, n = 3 technical repeats). (d) Hydrodynamic diameters of AuNCs prepared at different gold-to-GSH ratio to select AuNCs with sizes less than the kidney filtration threshold (5.5 nm) (Bars are mean of n = 3 measurements, mean values ± standard error).

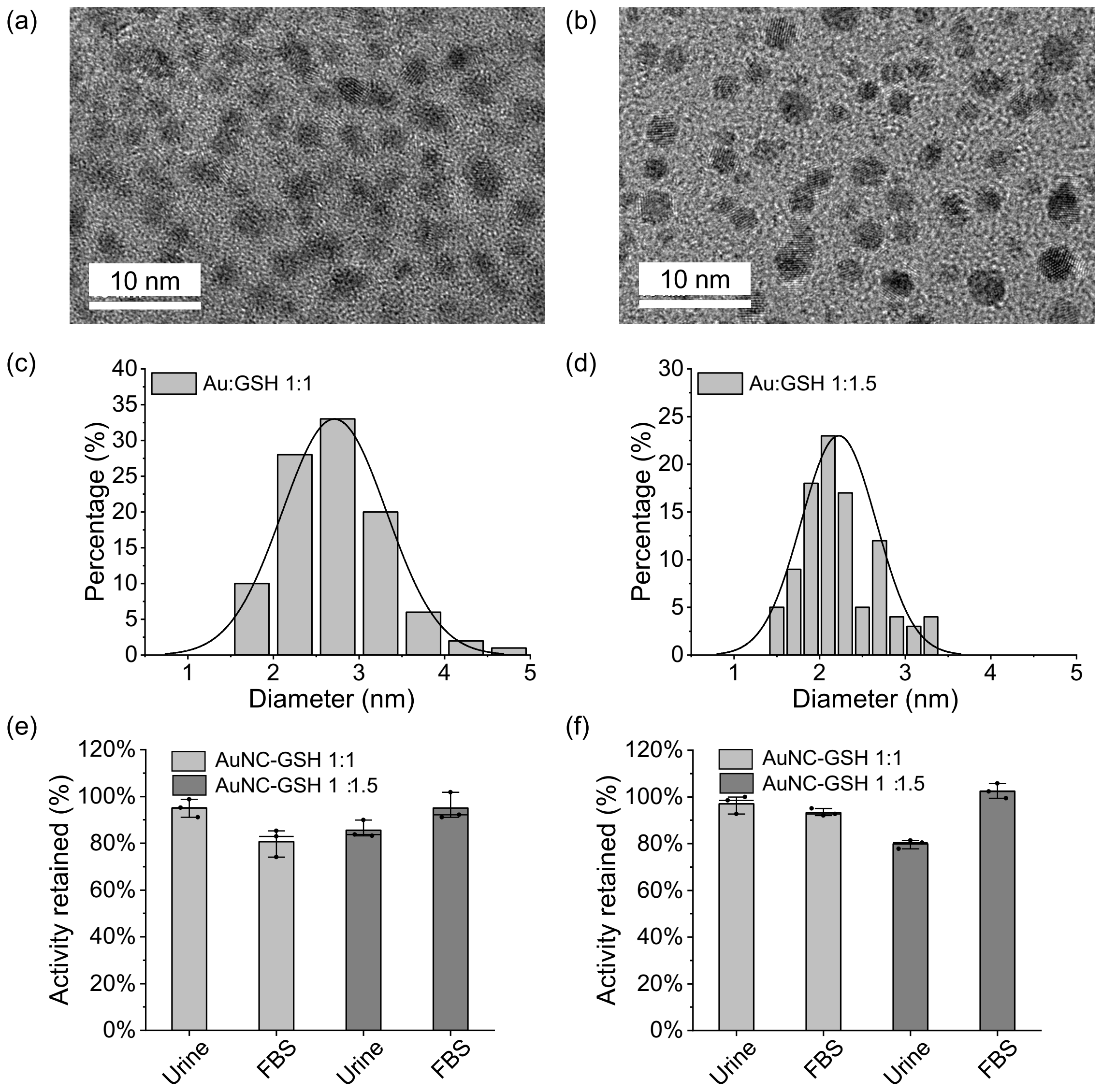

Supplementary Figure 3. TEM and stability characterisation of AuNC 1:1 and 1:1.5. (a, c) TEM image and the derived histogram of AuNC-GSH 1:1 (mean diameter = 2.7 ± 0.6 nm (s.d.), n = 100). (b, d) TEM image and the derived histogram of AuNC-GSH 1:1.5 (mean diameter = 2.2 ± 0.4 nm (s.d.), n = 100). (e, f) Retention of catalytic activity of both AuNCs in physiological environment such as urine (50 v/v %) and FBS (50 v/v %) compared to PBS (100 v/v %) after incubation for 1 hour (e) and 12 hours (f), respectively (mean values ± standard error, n = 3 technical repeats).

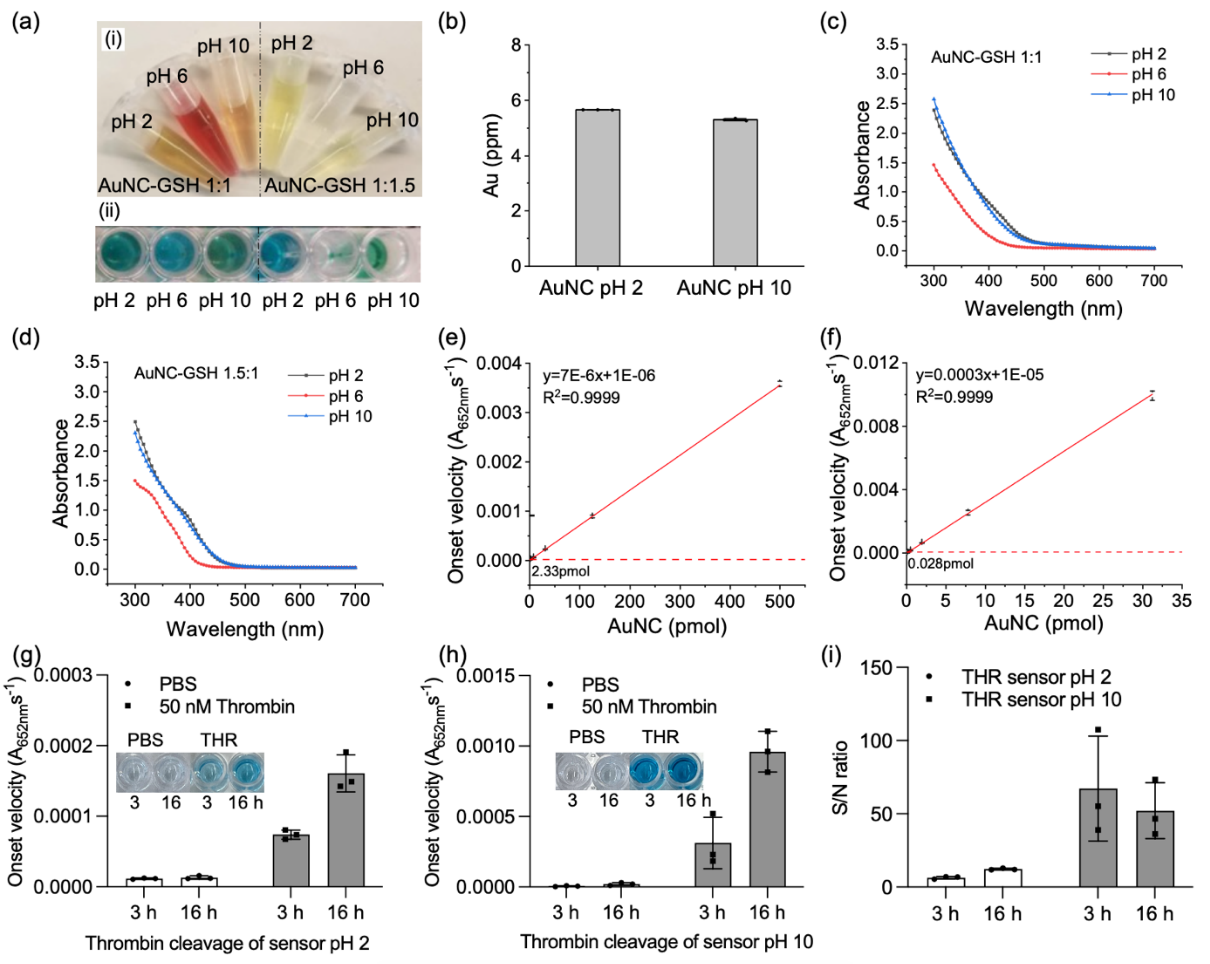

Supplementary Figure 4. Characterisation of AuNC 1:1 and 1:1.5 synthesised at different pH. (a) Photographs of (i) final products of all AuNCs synthesised at different pH yielding different colours and (ii) colourimetric readout after TMB oxidation assay for all AuNCs. (b) Inductively coupled plasma mass spectrometry (ICP-MS) measurement of gold content of AuNC-GSH 1:5 synthesised at pH 2 and 10 (mean values ± standard error, n = 3 technical repeats). This shows that the gold content from both AuNCs had similar amounts. (c, d) UV-Vis measurement of both AuNC-GSH 1:1 and 1:1.5 synthesised at different pH reaction conditions. This showed AuNCs synthesised at pH 6 might have produced a lower concentration and could account for the lower catalytic activity (N = 3 biological replicates, n = 1 technical repeat). The UV-Vis of AuNC-GSH in a 1:1.5 ratio synthesised at pH 2 and 10 exhibited similarities consistent with the gold content measured by ICP-MS (c). (e, f) The limit of detection (LoD, calculated as 3 times of standard deviations plus the mean value of the background) of AuNCs 1:1.5 from pH 2 and pH 10 reaction conditions, respectively. The catalytic activity was measured with the AuNCs diluted in synthetic urine (50 v/v %)

and evaluated by the onset velocity analysis from the oxidation of TMB substrate ($A_{652nm}s^{-1}$). The LoD of AuNCs 1:1.5 from pH 2 and 10 synthesis condition was 2.33 and 0.028 pmol (mean values ± standard error, n = 3 technical repeats), respectively, corresponding to 83-fold enhancement of catalytic activity, with the higher LoD value corresponding to the AuNCs of a related recent paper[2]. (g, h) Thrombin (THR) cleavage TMB assay on the filtrate after incubation of both THR sensors made of AuNC pH 2 (g) and AuNC pH 10 (h) for 3 and 16 hours of incubation. The signal from THR sensor pH 10 was 6x higher then THR sensor pH 2 (N = 3 biological replicates, n = 3 technical replicates). (i) The signal-to-noise (S/N) ratio (thrombin/PBS) for both sensor for 2 timepoints. THR sensor pH 10 revealed 4.3x and 10.5x higher S/N than THR sensor pH 2 (N = 3 biological replicates, n = 3 technical replicates).

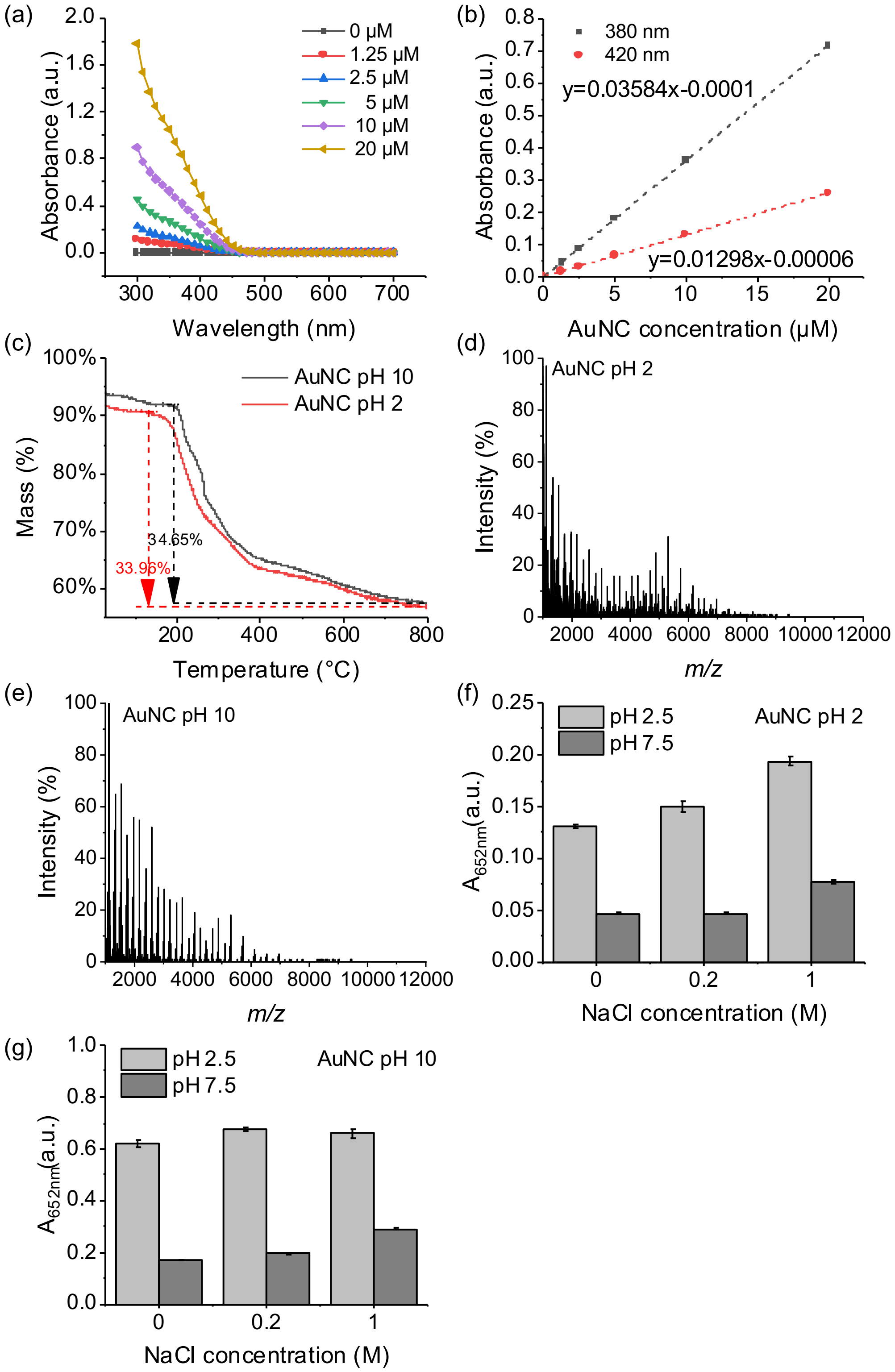

Supplementary Figure 5. Characterisation of the optimised AuNCs (1:1.5, pH 10). (a) UV-Vis measurement of the optimised AuNCs with serial dilution. Absorbance at 380/420 nm could be used for calibration of concentration for various batches. (b) The calibration curve for the absorbance of AuNCs at the wavelength of 380 nm and 420 nm from (a), allowed quantification of AuNC concentrations for batch comparisons (n

= 1 measurement). (c) Thermogravimetric analysis (TGA) of the previously developed and optimised AuNCs (pH 2 and 10). (d, e) Matrix-assisted laser desorption/ionisation time-of-flight (MALDI-TOF) mass spectrometry analyses of the previously studied and optimised AuNCs (pH 2 and pH 10). (f, g) Catalytic activity with varying NaCl concentration (0, 0.2, 1 M) after 1.5 min development using identical cluster concentrations (mean values ± standard error, n = 3 technical repeats).

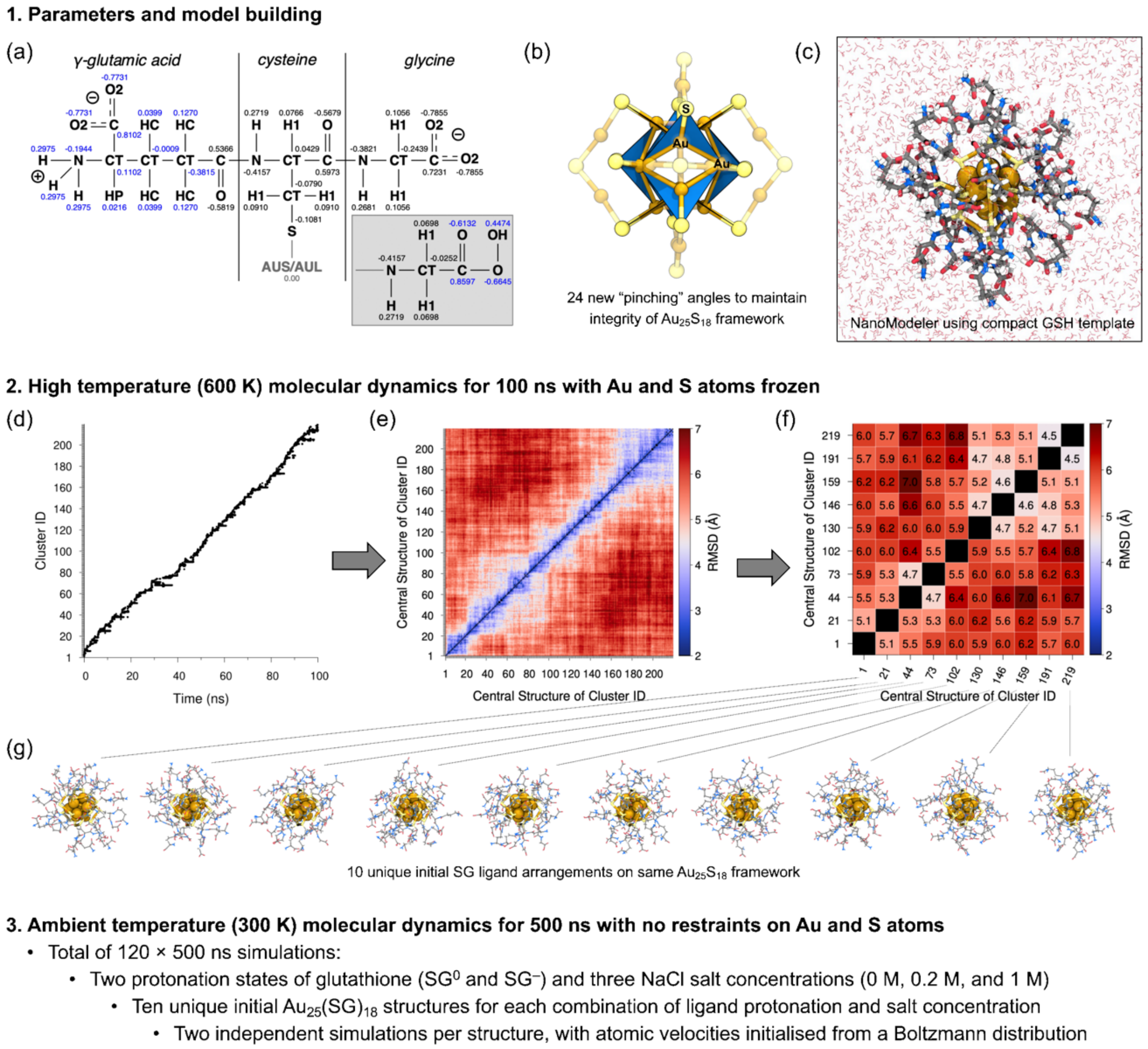

Supplementary Figure 6. Protocol and parameters used to generate $Au_{25}(SG)_{18}$ structures and perform molecular dynamics simulations. (a) Glutathione atom types and partial atomic charges, with optimised charges for N-terminal γ-glutamyl and protonated C-terminal glycine moieties in blue. (b) Visual depiction of the Au–Au–S angle parameters used to mitigate unphysical distortions. An equilibrium angle of 125° and harmonic force constant of 500 kJ/mol are employed, informed by equivalent parameters used for MD of an analogous Au_{25} NC[4]. (c) Initial solvated $Au_{25}(SG)_{18}$ model after building with NanoModeler[3] and compacted glutathione ligands. (d) RMSD cluster analysis after 100 ns of MD at elevated temperature of 600 K and frozen $Au_{25}S_{18}$ atoms to explore ligand conformational space. (e) Pairwise RMSD matrix comparing the ~200 central representative structures identified from each data cluster. (f) Condensed pairwise RMSD matrix for the ten most dissimilar structures. (g) Extracted structures shown separately to highlight diversity in ligand conformations.

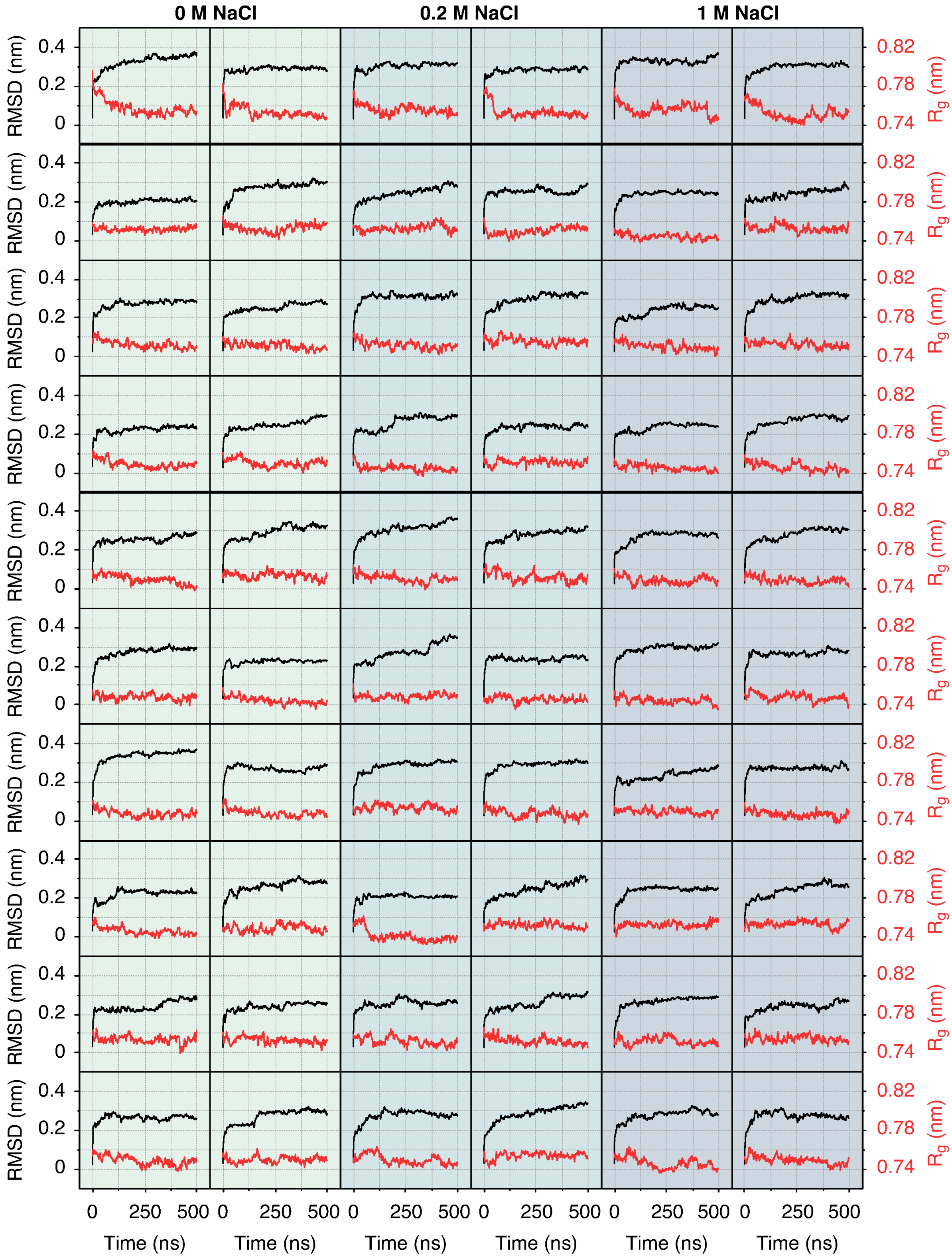

Supplementary Figure 7. RMSD and R_g plots for the low pH, $Au_{25}(SG^0)_{18}$ simulations. Each panel shows an independent trajectory, for the low (0 NaCl), medium (0.2 M NaCl) and high (1 M NaCl) salt concentrations. Plots are drawn using data every 5 ps but averaged over 500 frames to remove high frequency noise.

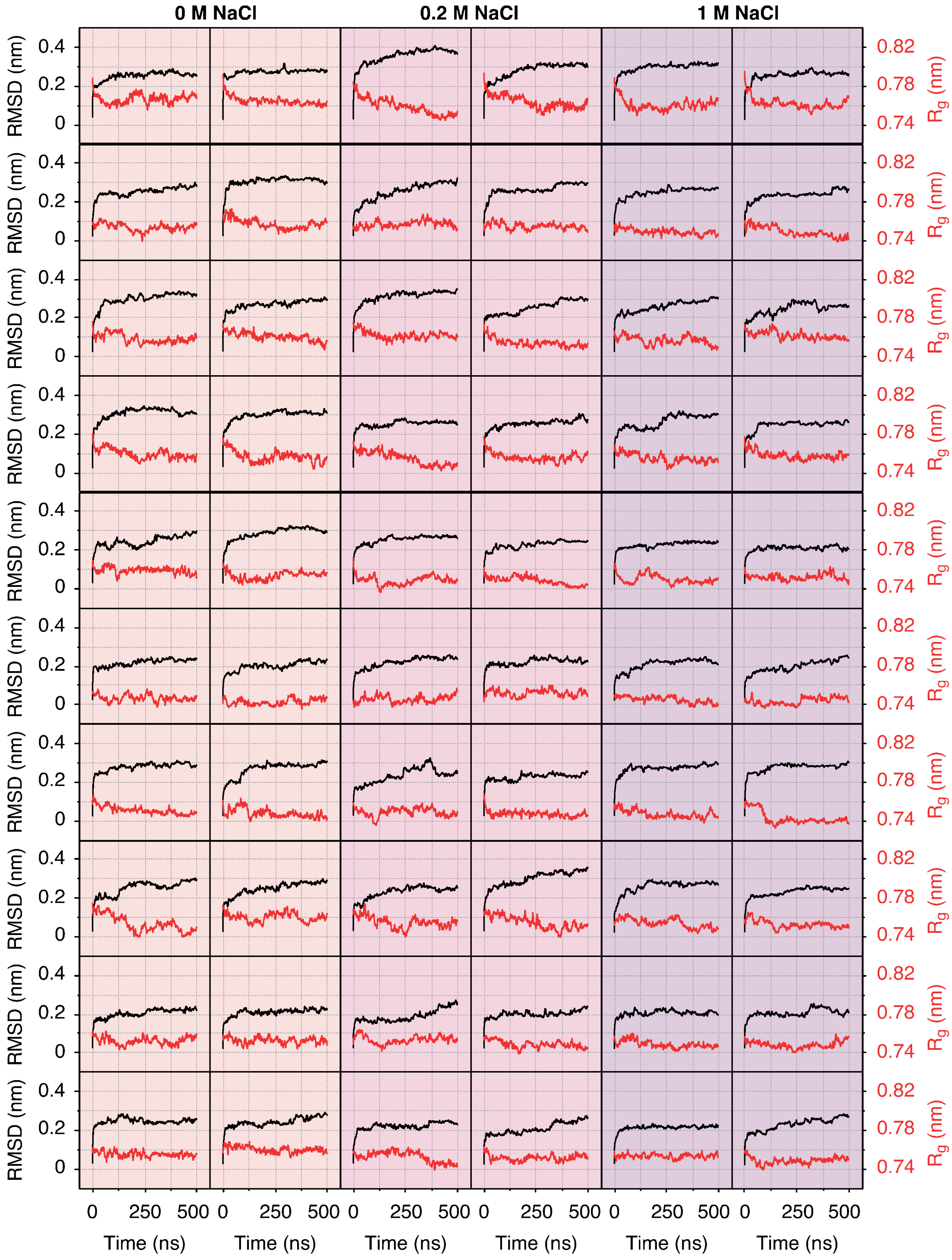

Supplementary Figure 8. RMSD and R_g plots for the low pH, $Au_{25}(SG^{-})_{18}$ simulations. Each panel shows an independent trajectory, for the low (0 NaCl), medium (0.2 M NaCl) and high (1 M NaCl) salt concentrations. Plots are drawn using data every 5 ps but averaged over 500 frames to remove high frequency noise.

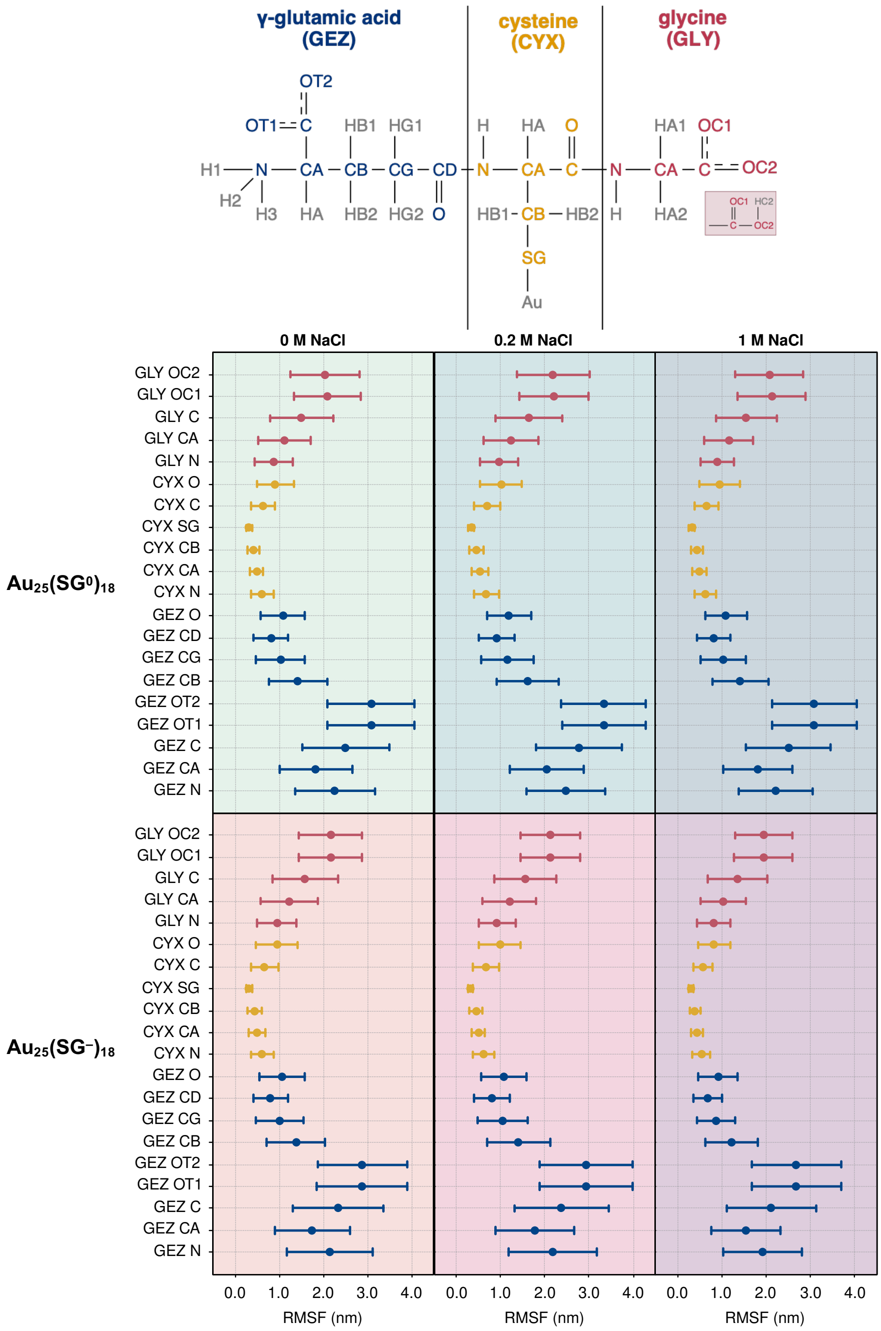

Supplementary Figure 9. Average atomic root-mean-square fluctuations (RMSF) of glutathione ligands' heavy atoms. Each panel displays the average atomic RMSF and standard deviation (errorbars) for the low (0 NaCl), medium (0.2 M NaCl) and high (1 M NaCl) salt concentration conditions. The averages are calculated using instantaneous configurations of $Au_{25}(SG)_{18}$, which are recorded every 5 ps during the final 300 ns of each of the 20 independent trajectories for each system. The RMSD data incorporates averages from all 18 individual ligands present on the nanoclusters. The structural formula of glutathione, showing all atom names, is displayed above.

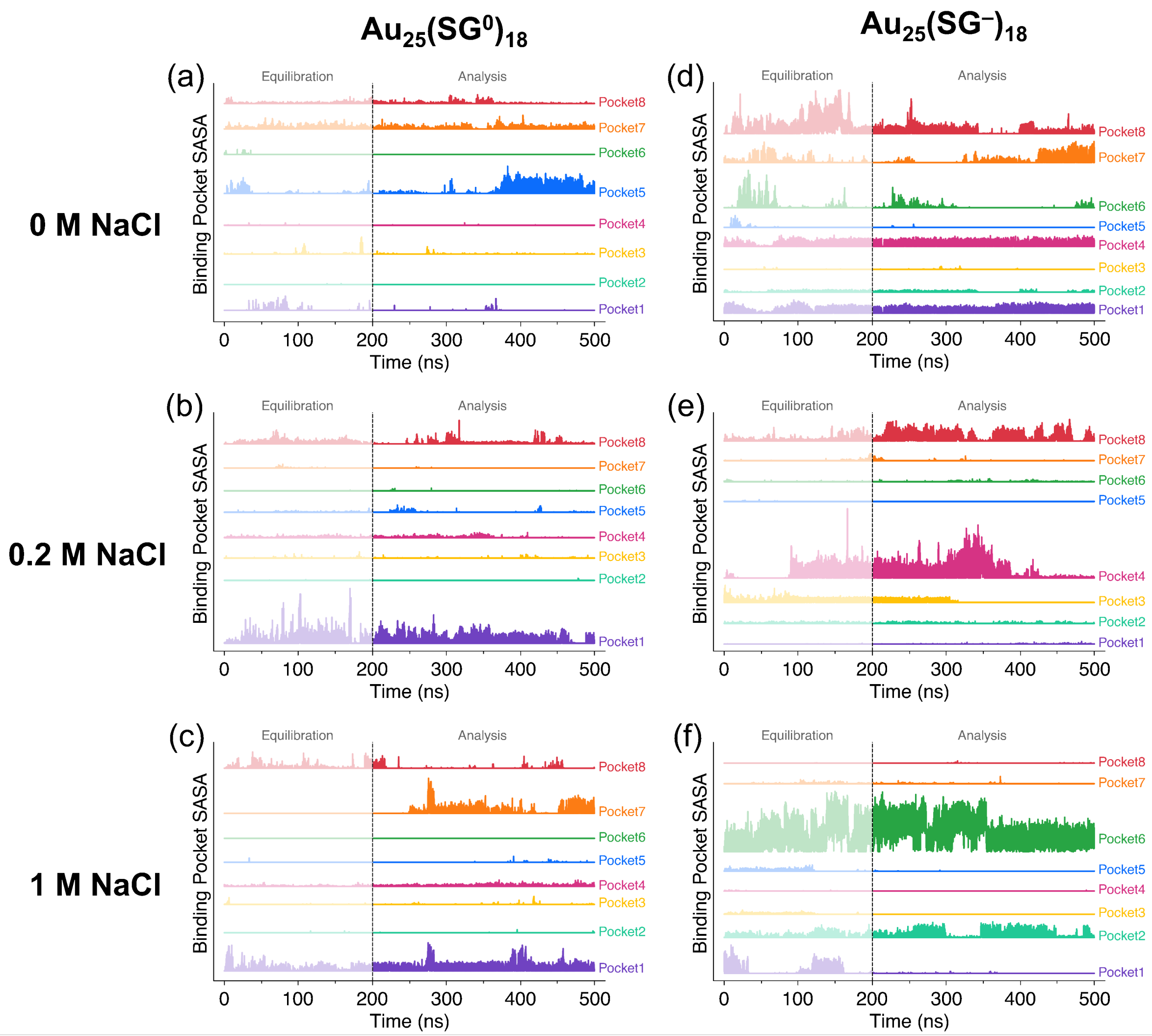

Supplementary Figure 10. SASA for each of the eight binding pockets over time, for representative trajectories of each system. SASA values greater than 0 nm^2 signify an individual pocket is accessible to small molecules, such as such as H_2O, H_2O_2, and TMB. The presented plots are chosen to illustrate a single trajectory from the ensemble of 20 trajectories per system. The y-scale of each plot is relative to the largest measured binding pocket SASA over the analysis period: (a) Pocket5 (blue) = 9.2 nm^2 at 383 ns; (b) Pocket1 (purple) = 13.9 nm^2 at 283 ns; (c) Pocket7 (orange) = 13.8 nm^2 at 276 ns; (d) Pocket8 (red) = 17.5 nm^2 at 253 ns ; (e) Pocket4 (pink) = 29.4 nm^2 at 343 ns ; (f) Pocket6 (green) = 31.4 nm^2 at 214 ns. A graphic animation illustrating the dynamic opening and closing of an accessible active site can be found in Supplementary Movie 1. Colours for the plots match those in Figure 2f (main text).

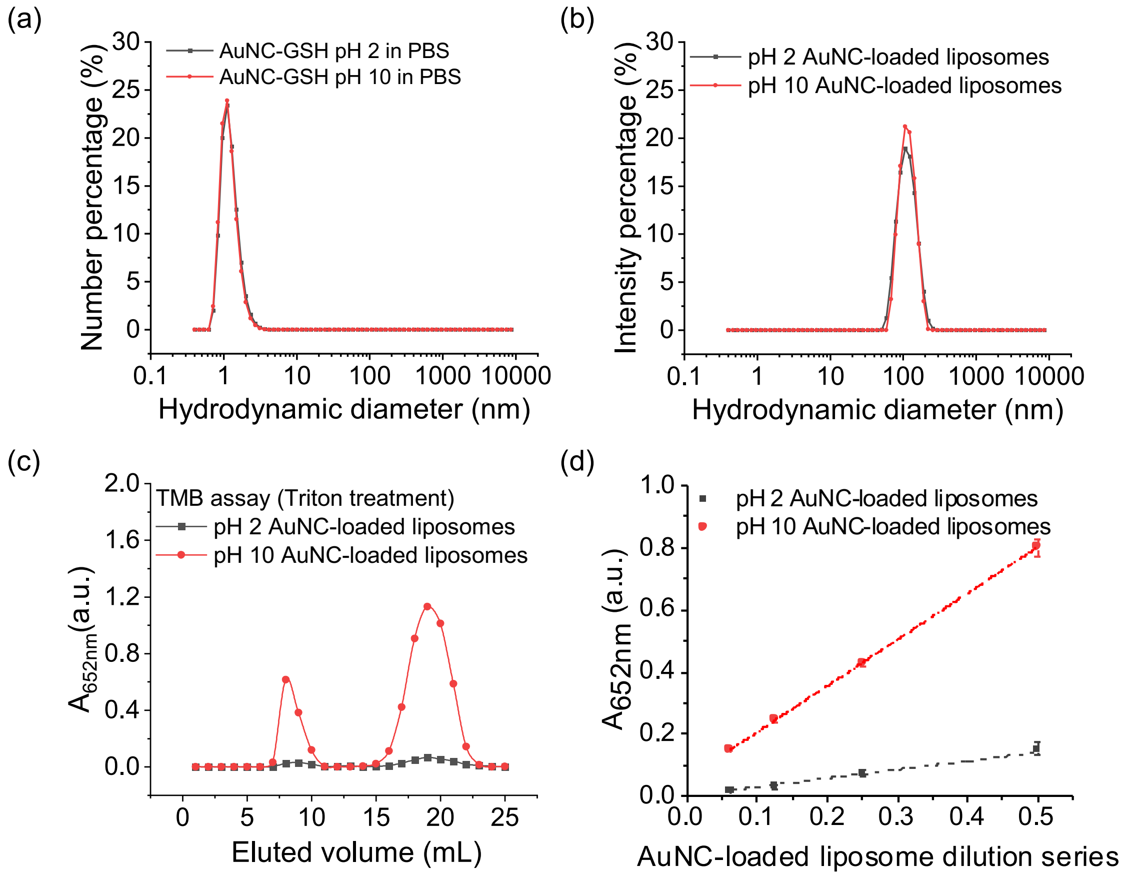

Supplementary Figure 11. Characterisation of AuNC pH 2 and pH 10 loading into liposomes. (a) Number distribution of AuNC pH 2 and pH 10 by DLS. Plot shows that the size of both AuNCs ~2 nm (curves are mean n = 2 technical repeats). (b) Intensity distribution of liposomes loaded with AuNC pH 2 and pH 10 by DLS. Plot shows the size of both liposomes ~120 nm (curves are mean of n = 3 technical repeats). (c) TMB oxidation assay on the eluted fractions of both liposomes through size exclusion chromatography (SEC) with subsequent Triton X-100 treatment to lyse the samples. This shows that both the liposome fractions and unloaded AuNCs fractions exhibited significantly higher catalytic activity for the samples with the optimised AuNCs pH 10, with a similar trend observed when the assay was performed with Triton X-100 treatment (n = 1 measurement). (d) Calibration curve of both liposomes loaded with AuNCs pH 2 and 10. This could be used for the quantification of released AuNCs (mean values ± standard error, n = 2 technical repeats).

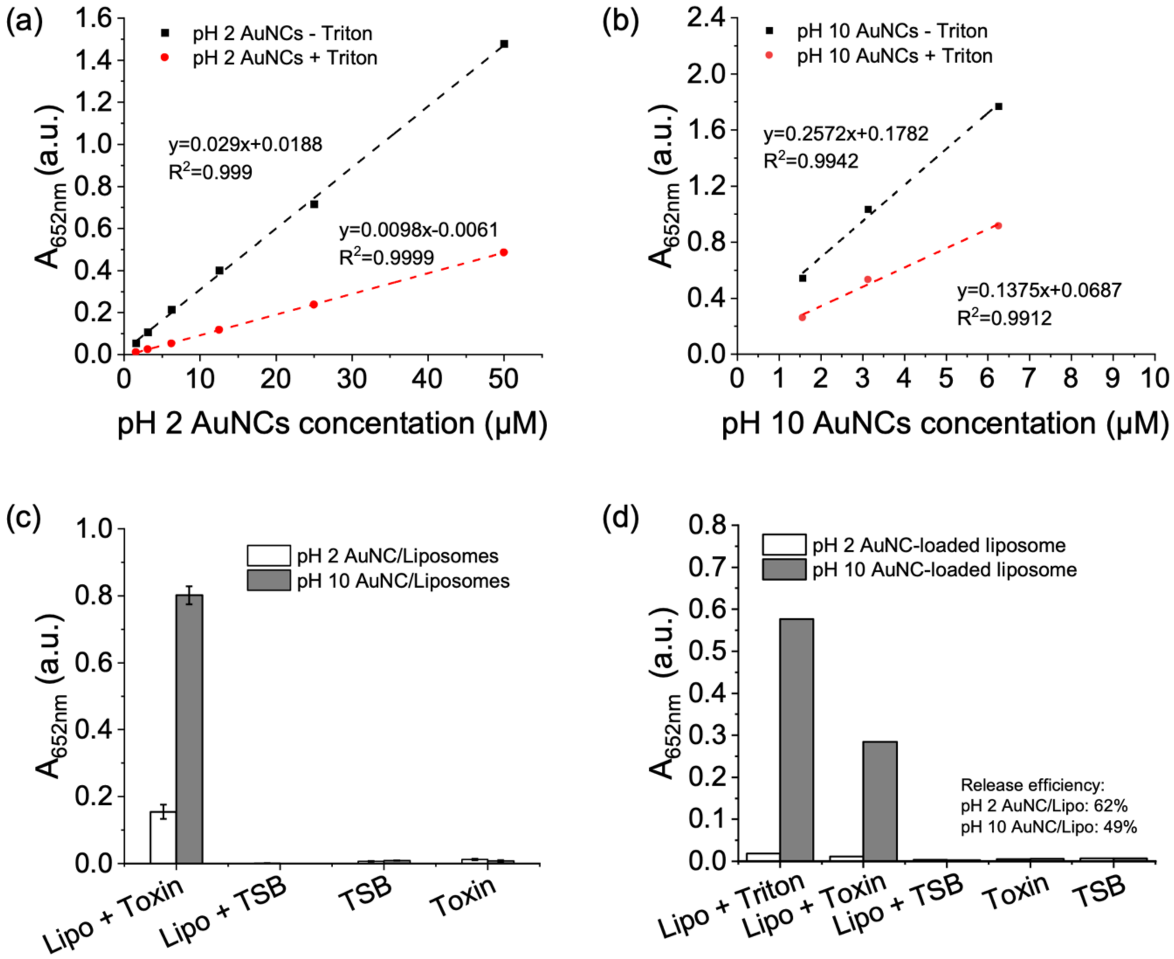

Supplementary Figure 12. Calibration curves for AuNCs and toxin release data for liposomal sensors. (a, b) Calibration curves for TMB assay endpoint measurements using AuNCs pH 2 and 10 in PBS or 10 v/v % Triton X-100. TMB assay endpoint for AuNCs pH 2 was plotted at 100 seconds and for AuNCs pH 10 at 80 s. This provided a reference for the accurate calculation of the amount of both AuNCs when measured in an unknown sample (n = 1 technical repeat). (c, d) TMB assay endpoint measurements after incubation of liposomes with toxin or TSB as control or 10 v/v % Triton X-100 as full release control for 2 h, Amicon filtered and TMB assay performed on filtrates from two invidual experiments (n = 2 technical repeats for (c) and n = 1 for (d) with the release efficiency).

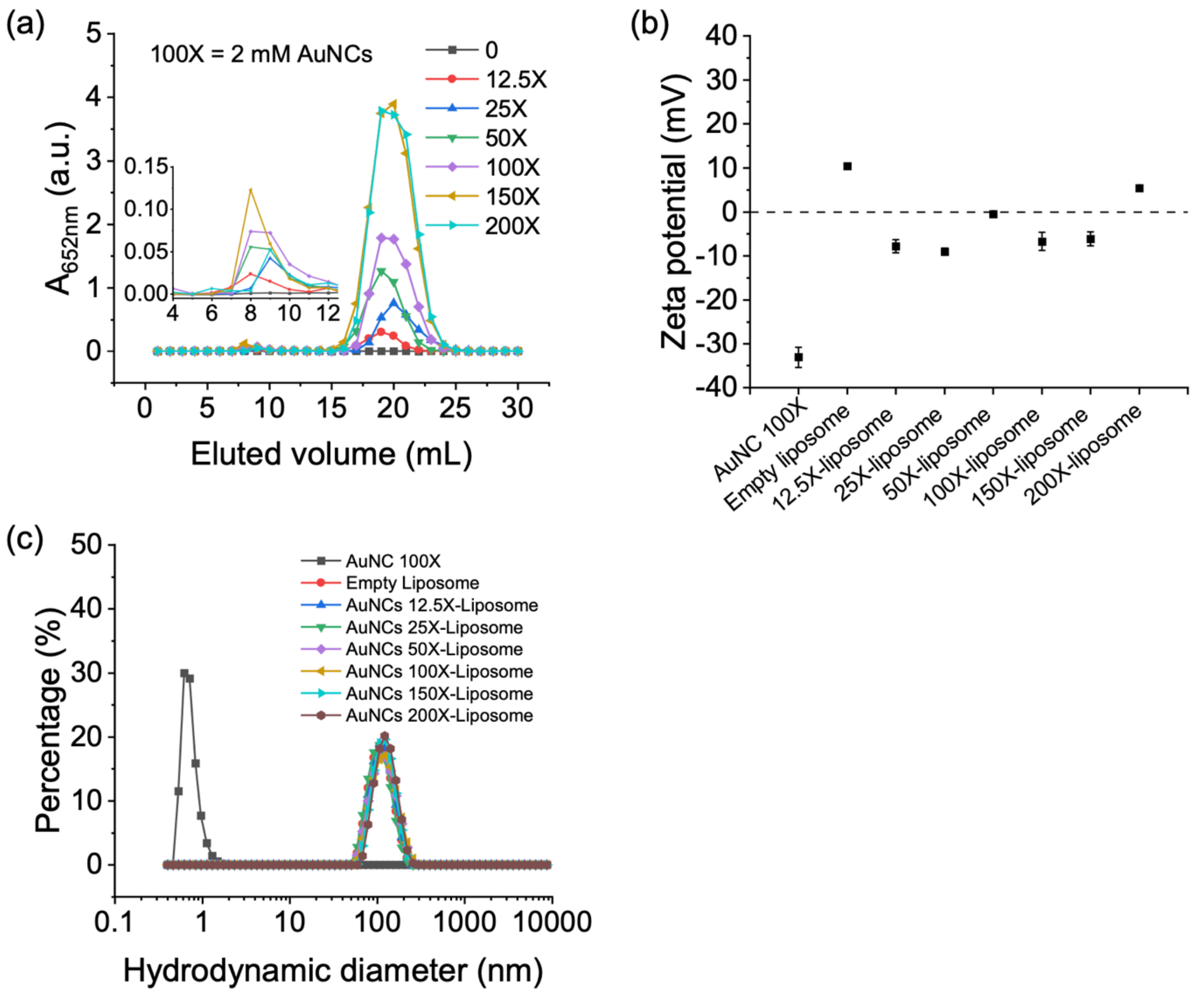

Supplementary Figure 13. Optimisation of AuNC concentration for encapsulation in liposomes. (a) TMB oxidation assays on the eluted fractions in PBS after SEC of AuNC-loaded liposomes with different concentrations of AuNCs (12.5X corresponds to 0.25 mM during loading, and 200X to 4 mM). Larger liposomes were eluted faster (first collection peak, zoom insert) than AuNCs (second, much larger peak). All fractions were diluted 20X for TMB measurement (n = 1 measurement). (b) Zeta potential measurements of AuNCs, bare BSM:CH (50:50 w/w) liposomes and the liposomes loaded with different concentrations of AuNCs (mean values ± standard error, n = 3 technical repeats). (c) DLS size distributions of AuNCs (number distribution), bare liposomes and AuNC-loaded liposomes (intensity distributions) (curves are mean of n = 3 technical repeats).

Supplementary Table 2. ICP-MS measurement of gold contents in AuNC-loaded liposomes for encapsulation efficiency calculation. 12.5X corresponds to 0.25 mM, going up to 200X, which corresponds to 4 mM AuNCs used during loading.

AuNC	ppm 100X dil.	STDEV	AuNC-liposome	ppm	STDEV	Encapsulation efficiency (%)
AuNC 0 (PBS)	0.0341	0.0112	Empty liposomes	0.0434	0.0311	1.27%
AuNC 12.5X	2.12	0.006	AuNC 12.5X-loaded liposomes	6.992	0.04	3.30%
AuNC 25X	3.645	0.028	AuNC 25X-loaded liposomes	11.12	0.03	3.05%
AuNC 50X	9.415	0.069	AuNC 50X-loaded liposomes	20.31	0.11	2.16%
AuNC 100X	14.36	0.12	AuNC 100X-loaded liposomes	36.3	0.25	2.53%
AuNC 150X	25.57	0.09	AuNC 150X-loaded liposomes	25.2	0.28	0.99%
AuNC 200X	31.04	0.07	AuNC 200X-loaded liposomes	36.92	0.17	1.19%

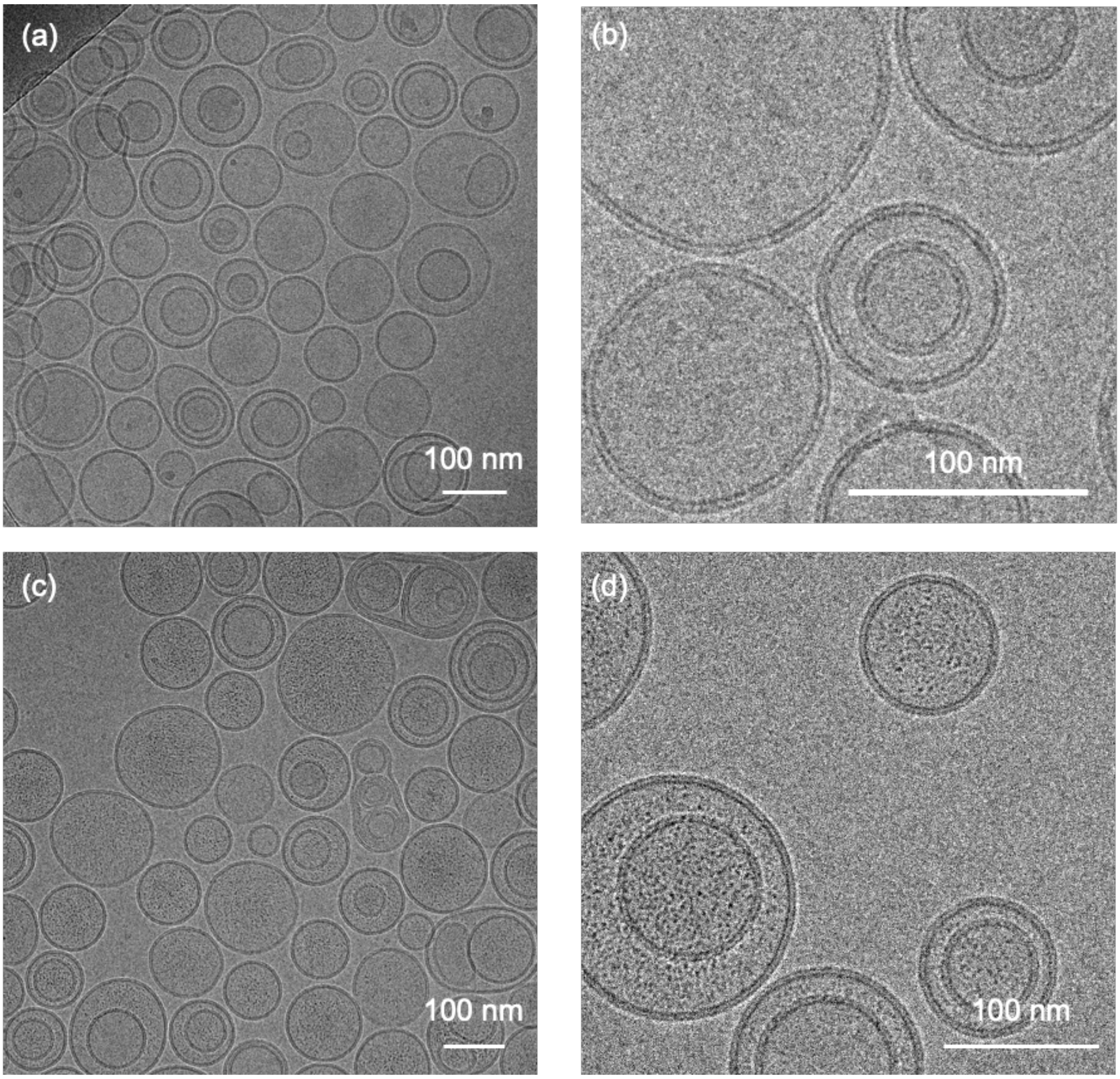

Supplementary Figure 14. Cryo-TEM images of empty liposome control and AuNC-loaded liposomes. (a, b) Empty liposome control (BSM:PC:CH). (c, d) AuNC-loaded liposomes (BSM:PC:CH), showing the successful core loading of AuNCs (dark dots). Scale bars = 100 nm.

Supplementary Table 3. Liposome compositions used (brain sphingomyelin (BSM), 1-palmitoyl-2-oleoyl-glycero-3-phosphocholine (16:0-18:1 PC), cholesterol (CH), 1,2-dioleoyl-3-trimethylammonium-propane (DOTAP) with 1 mol % of 1,2-distearoyl-sn-glycero-3-phosphoethanolamine-N-[methoxy(polyethylene glycol)-2000] (DSPE-PEG-2K). All % are mass %.

**Lipid formulation**	BSM:CH	PC:CH	BSM:PC:CH /BPC	BPC+1% DOTAP	BPC+5% DOTAP	BPC+10% DOTAP	BPC+25% DOTAP
BSM	50%	N/A	33.3%	33%	31.7%	30%	25%
PC	N/A	50%	33.3%	33%	31.7%	30%	25%
CH	50%	50%	33.3%	33%	31.7%	30%	25%
DSPE-PEG-2k (mol% to lipids)	1%	1%	1%	1%	1%	1%	1%
DOTAP	N/A	N/A	N/A	1%	5%	10%	25%

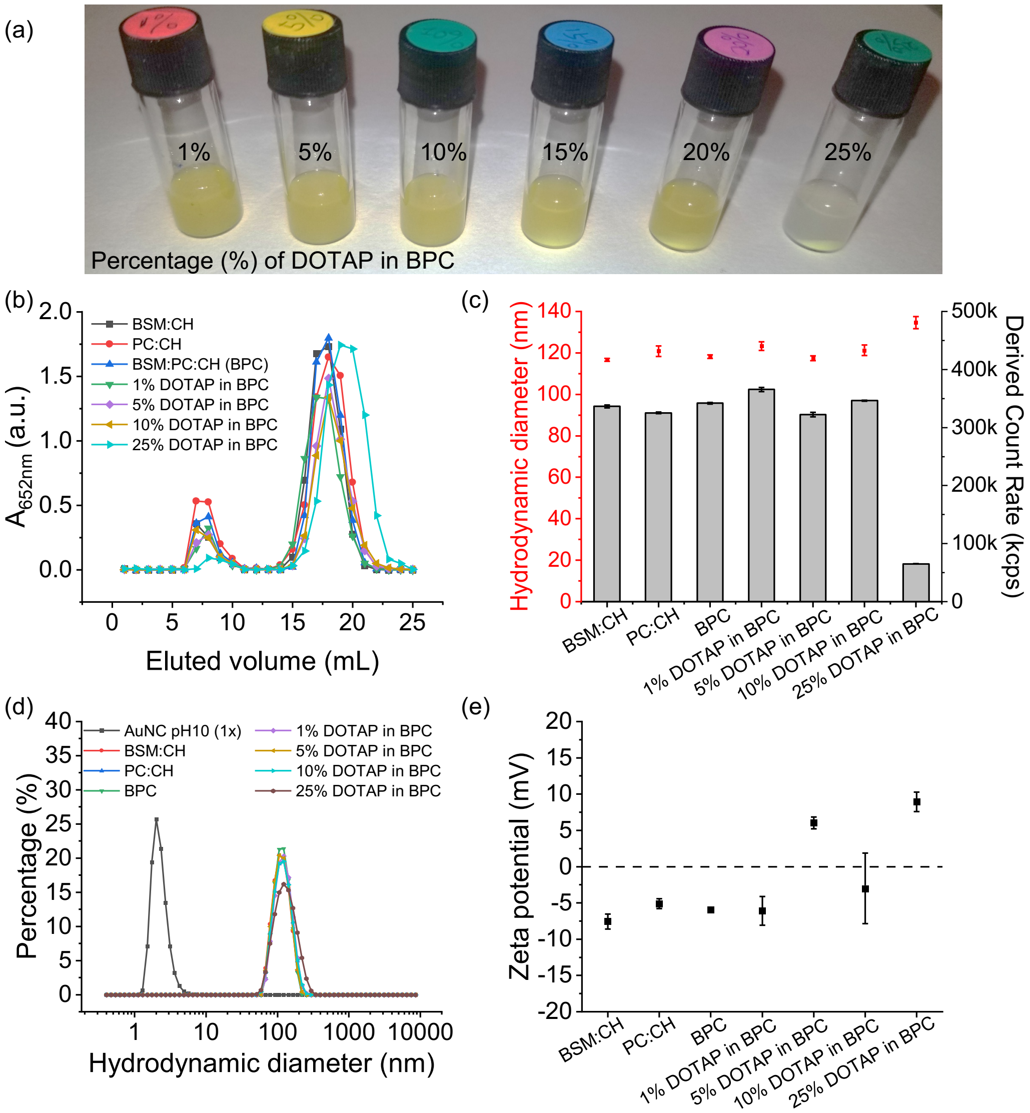

Supplementary Figure 15. Optimisation and characterisation of liposome compositions using DOTAP lipid. (a) Photograph of all liposome solutions containing different percentages of DOTAP in BPC formulation. Images show that aggregation occurred when adding more than 15 wt. % of DOTAP. (b) TMB oxidation assay on the SEC elution fractions for all liposomes. Notably, liposomes with 25 wt. % DOTAP exhibited a much lower absorbance signal in the liposome fractions, which corresponded to the observed aggregations (n = 1 technical repeat). (c) Hydrodynamic diameter and derived count rate measurements of all liposome samples by DLS demonstrates similar size and corresponding concentration (mean values ± standard error, n = 3 technical repeats). Liposomes with 25 % DOTAP showed a lower derived count rate, consistent with the aggregations in the photograph (a) and lower TMB signal (b) (n = 1 measurement). (d) DLS size distribution of AuNCs (number distribution) and all AuNC-loaded liposomes (intensity distribution) (curves are mean

of n = 3 technical repeats). (e) Zeta potential measurements of all different formulations of liposomes loaded with AuNCs. The addition of cationic DOTAP lipids could lead to a positive charge in the AuNC-loaded liposomes (n = 3 technical repeats).

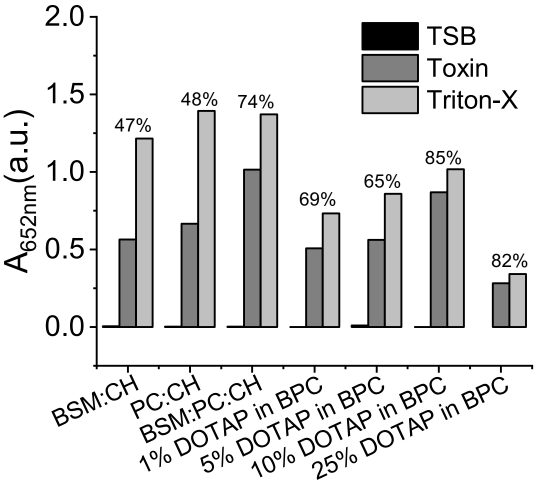

Supplementary Figure 16. Toxin release efficiency comparison for all liposomal sensor versions. TMB assay endpoint absorbance after incubating AuNC-liposomes in TSB, TSB + toxin, PBS + 10 v/v % Triton X-100 and separating liberated AuNCs by centrifugal filtration and analysing the filtrates. Liposomes with 10 wt. % and 25 wt. % DOTAP had an optimal toxin release efficiency followed by liposomes (BSM:PC:CH) without DOTAP. Considering the negative surface charge, in the absence of aggregation, as well as the overall higher catalytic activity even at a slightly lower release efficiency, the BPC liposome sensor (BSM:PC:CH) without DOTAP was the lead formulation that was selected for the subsequent experiments (n = 1 technical repeat).

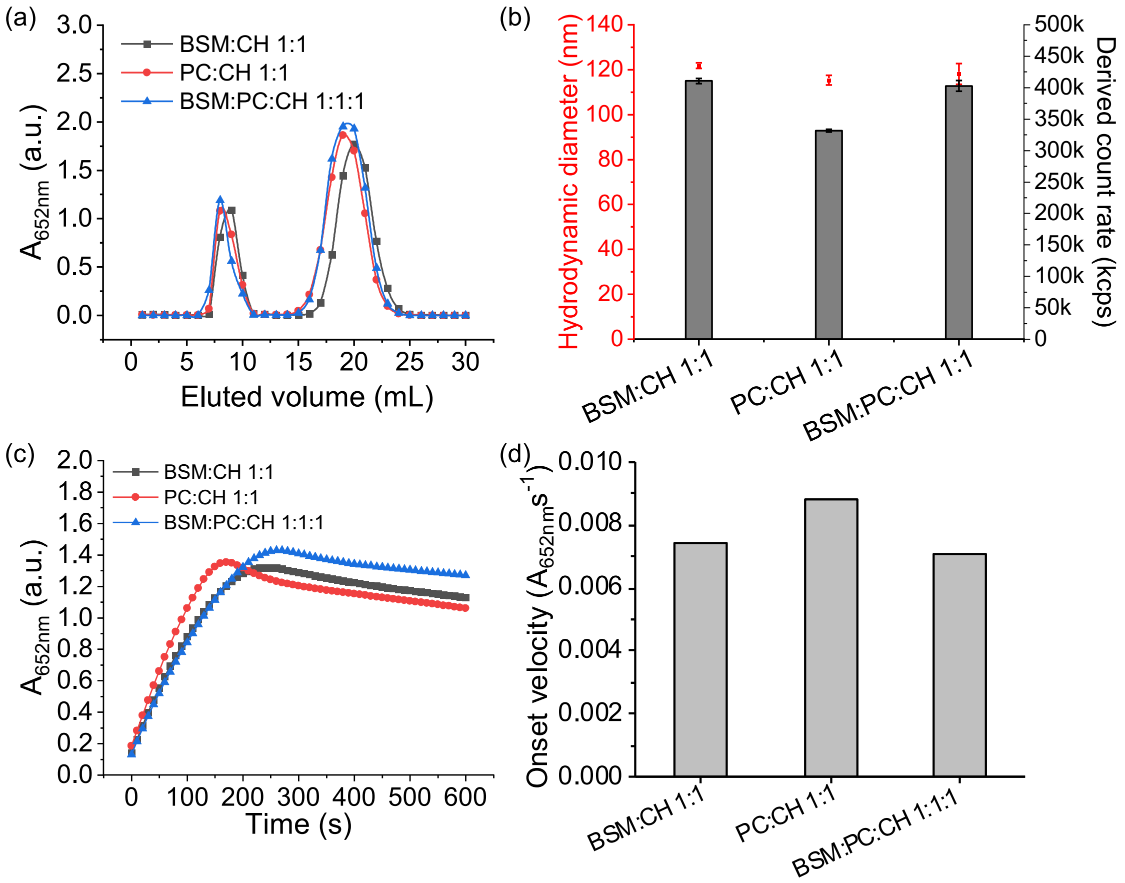

Supplementary Figure 17. Characterisation of AuNC loading in liposomes with different lipid compositions. (a) TMB oxidation assays on SEC elution fractions of three different formulations of liposomes. The first 10 mL of fractions were diluted 2X, and the rest 20 mL of free unloaded AuNCs were diluted 20X. TMB assay was conducted on diluted fractions (100 μL) using TMB (50 μL) and H_2O_2 (2 M, 50 μL) (n = 1 technical repeat). (b) Hydrodynamic diameter (intensity distribution) and derived count rate measurements of the three liposome samples by DLS demonstrates similar size and concentration, which was further confirmed by nanoparticle tracking analysis (NTA, Supplementary Table 4) (mean values ± standard error, n = 3 technical repeats). (c, d) Characterisation of the catalytic activity of the three AuNC-liposomes (n = 1 measurement). The increase in TMB absorbance over time for three liposomes was measured on a plate reader and the onset velocity (d) was derived from the absorbance kinetics (c) (n = 1 technical repeat).

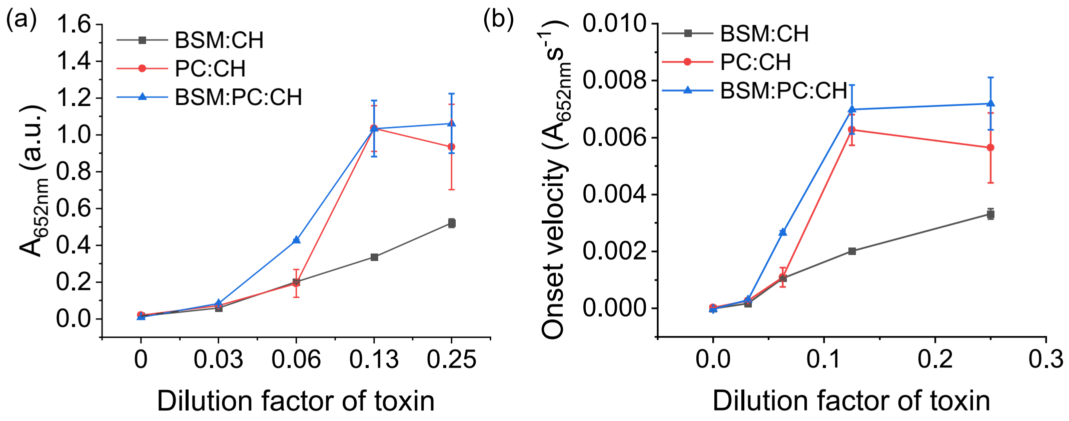

Supplementary Figure 18. LoD comparison for three AuNC-liposome compositions after incubation with a toxin dilution series. The end point of absorbance (a) and the derived onset velocity (b) determined via the filtrates after incubation of the different liposomal sensors with various dilutions of *S. aureus* SH1000 overnight culture supernatant containing secreted toxins and centrifugal filtration to separate intact sensors from released AuNCs (mean values ± standard error, N = 2 biological replicates, n = 1 technical measurement).

Supplementary Table 4. NTA characterisation of all three liposome samples.

**NTA measurement**	Mean size	SD	Normalised N	n=N/NA (mol)
BSM:CH	118.5 nm	27.3 nm	2.23e+013 +/- 5.02e+07	3.703e-11
PC:CH	123.1 nm	27.3nm	2.05e+013 +/- 1.14e+08	3.404e-11
BSM:PC:CH	115.6 nm	23.8nm	1.71e+013 +/- 2.12e+08	2.8395e-11

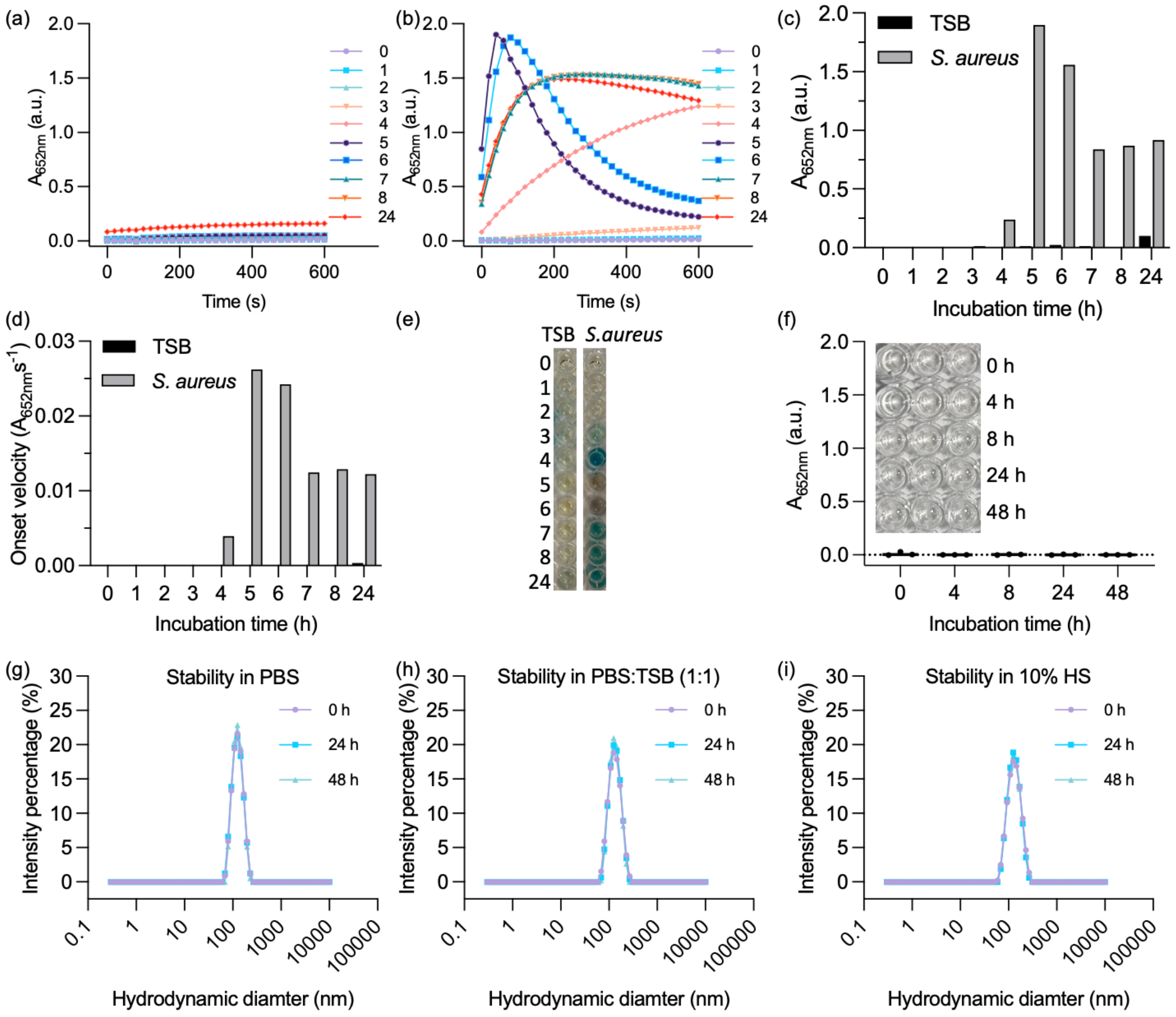

Supplementary Figure 19. Response performance of AuNC-loaded liposomes to toxin by co-incubation with *S. aureus* SH1000. (a, b) The measurement of absorbance over time for TMB assay on the filtrates collected after various time periods after incubation of AuNC-loaded liposome with TSB and TSB + bacteria. (c) Absorbance measurement at the endpoint of 140 seconds from (a, b) (n = 1 technical repeat). (d) Onset velocities calculated from (a, b). (e) Photographs show the final TMB assay results from (a-d); Incubation time varies from 0 to 24 hours (N = 1 biological replicate, n = 1 technical repeat). (f) Stability of liposomal sensor in TSB from 0 to 48 hours. The filtrates were collected after incubating liposomal sensor in TSB for 0 to 48 hours, followed by the TMB assay. The insert photograph shows no blue colour change after TMB colourimetric reaction (N = 3 biological replicates, n = 3 technical repeats). (g-i) DLS measurements of liposomes in PBS (g), PBS:TSB 1:1 (h), and 10 v/v% HS (human serum) (i), after up to 48 hours of incubation (curves are mean of n = 3 technical measurements).

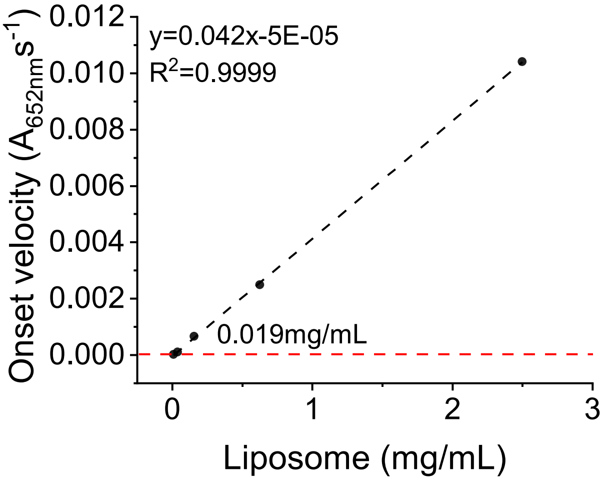

Supplementary Figure 20. The calibration curve of the BSM:PC:CH liposomal sensor. The calibration curve of the AuNC-loaded liposomes. The liposomal sensor was diluted in urine (50/50 v/v) to get different concentration (mg/mL) (n = 1 technical repeat).

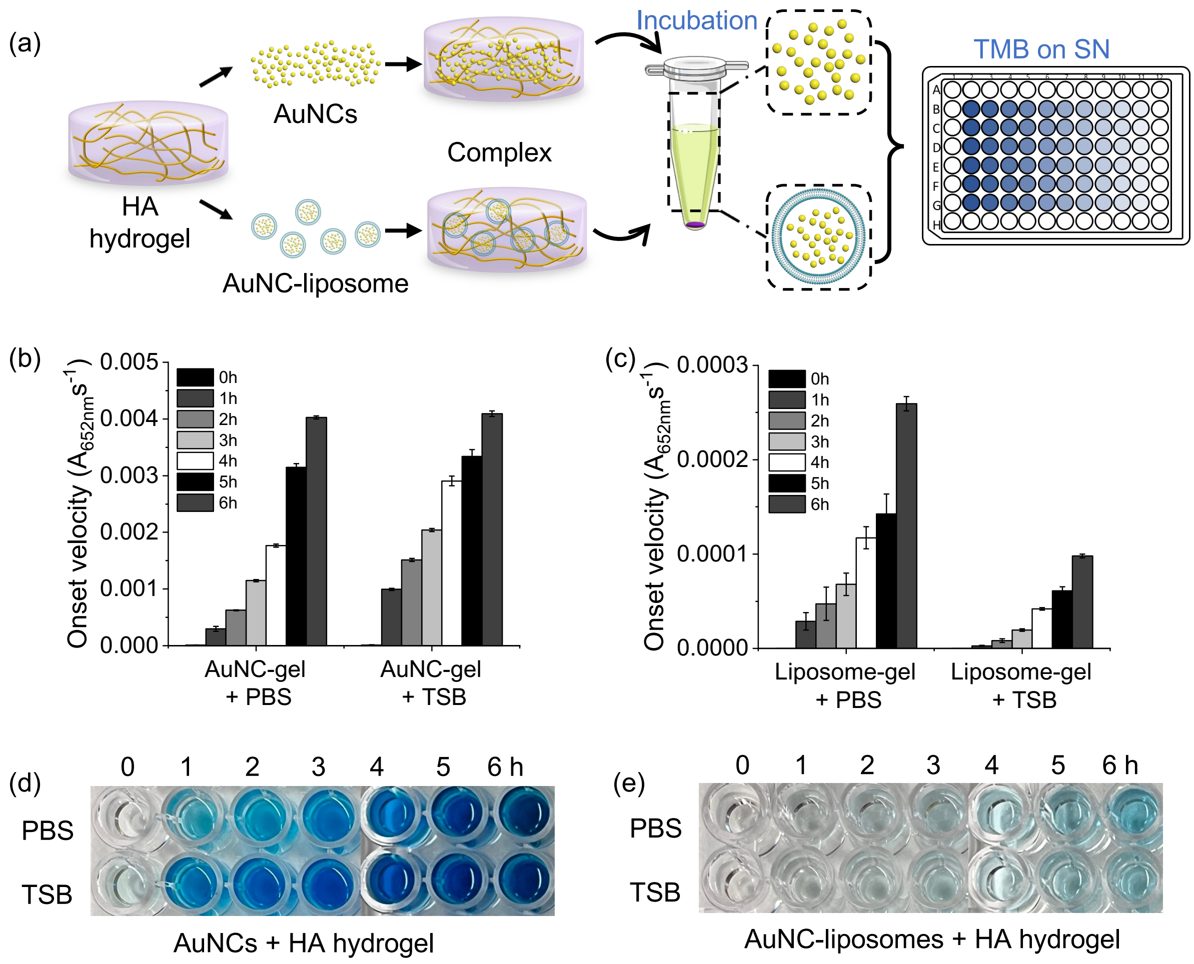

Supplementary Figure 21. Evaluation of AuNCs and AuNC-liposome retention within HA hydrogels. (a) Schematic of incubation of HA hydrogels with AuNCs and AuNC-loaded liposomes and evaluation of the release out of the gel. The supernatants, containing the released AuNCs or AuNC-liposomes, were collected after incubation of these mixtures (HA hydrogel with AuNCs or AuNC-liposomes), followed by TMB assay. (b-c) Onset velocity calculated from catalytic activity measurements (mean values ± standard error, n = 3 technical repeats). (d-e) photographs of the wells after collecting supernatants from AuNC- or AuNC-liposome-loaded HA hydrogels incubated samples.

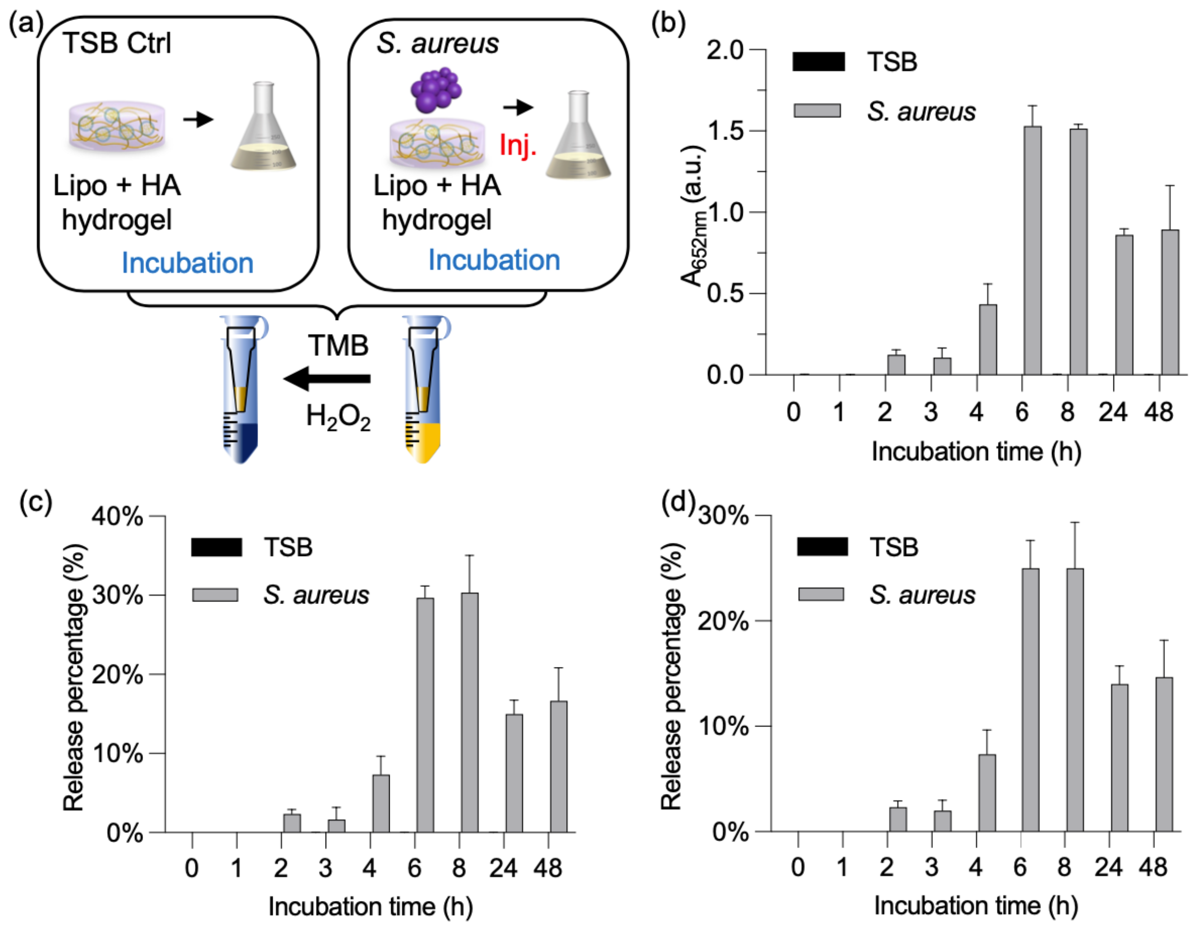

Supplementary Figure 22. Bacteria-triggered release of AuNCs from liposomal sensors within HA hydrogels. (a) Schematic of the *in vitro* procedure mimicking *in vivo* experiment. (b) TMB assay (absorbance measurement) on filtrates after incubation of the liposomal sensor mixed with HA gel in TSB +/- bacteria, showing absorbance of TMB reaction upon release of AuNCs. The incubation time varied from 0 to 48 hours (mean values ± standard error, N = 3 biological replicates, n = 3 technical repeats). (c, d) Release efficiency calculated from onset velocity (Figure 6d) and absorbance (b) (mean values ± standard error, N = 3 biological replicates, n = 3 technical repeats).

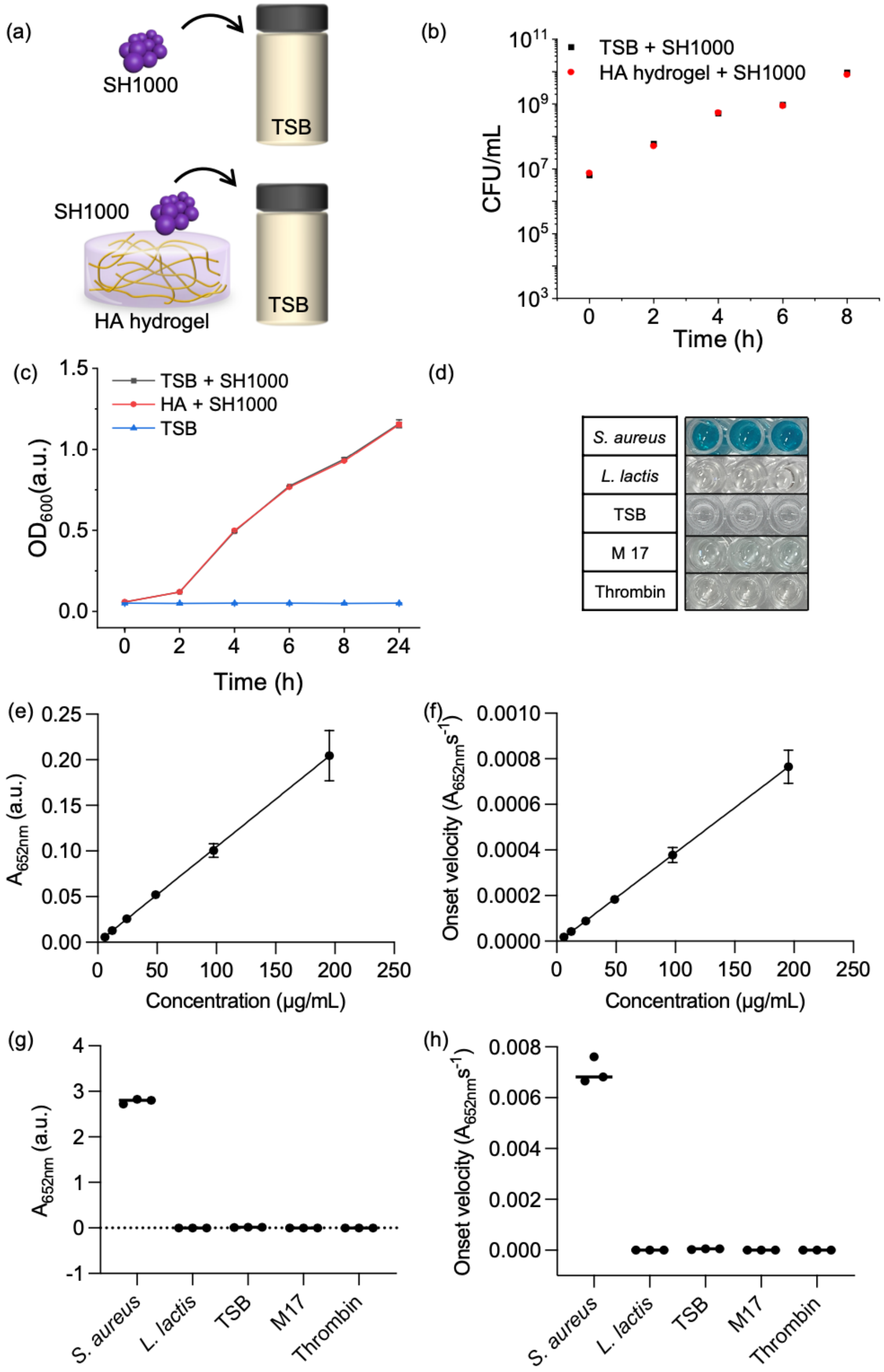

Supplementary Figure 23. Bacteria growth in presence of HA hydrogels and responses of the liposomal sensor to *S. aureus* and *L. lactis***.** (a) Schematic of

incubation of *S. aureus* SH1000 strain with and without HA hydrogel. (b, c) CFU measurements (n = 1 measurement) and absorbance at 600 nm measurements (n = 3 measurements) of the samples from SH1000 incubation with and without HA hydrogel demonstrating the gel had no effect on bacteria growth (n = 3 technical repeats). (d) Photographs of TMB results for (g, h). (e, f) The calibration curve of the liposomal sensor by TMB assay, calculating the onset velocity (e) and measuring the endpoint absorbance (f) (N = 3 biological replicates, n = 1 technical repeat). (g, h) TMB colourimetric readout results on the filtrates after Amicon filtration post 3-hour incubation of the liposomal sensor with supernatants from *S. aureus* and *L. lactis*, thrombin and the media controls (TSB and M 17 media), showing the absorbance (g), onset velocity (h) (N = 3 biological replicates, n = 3 technical repeats).

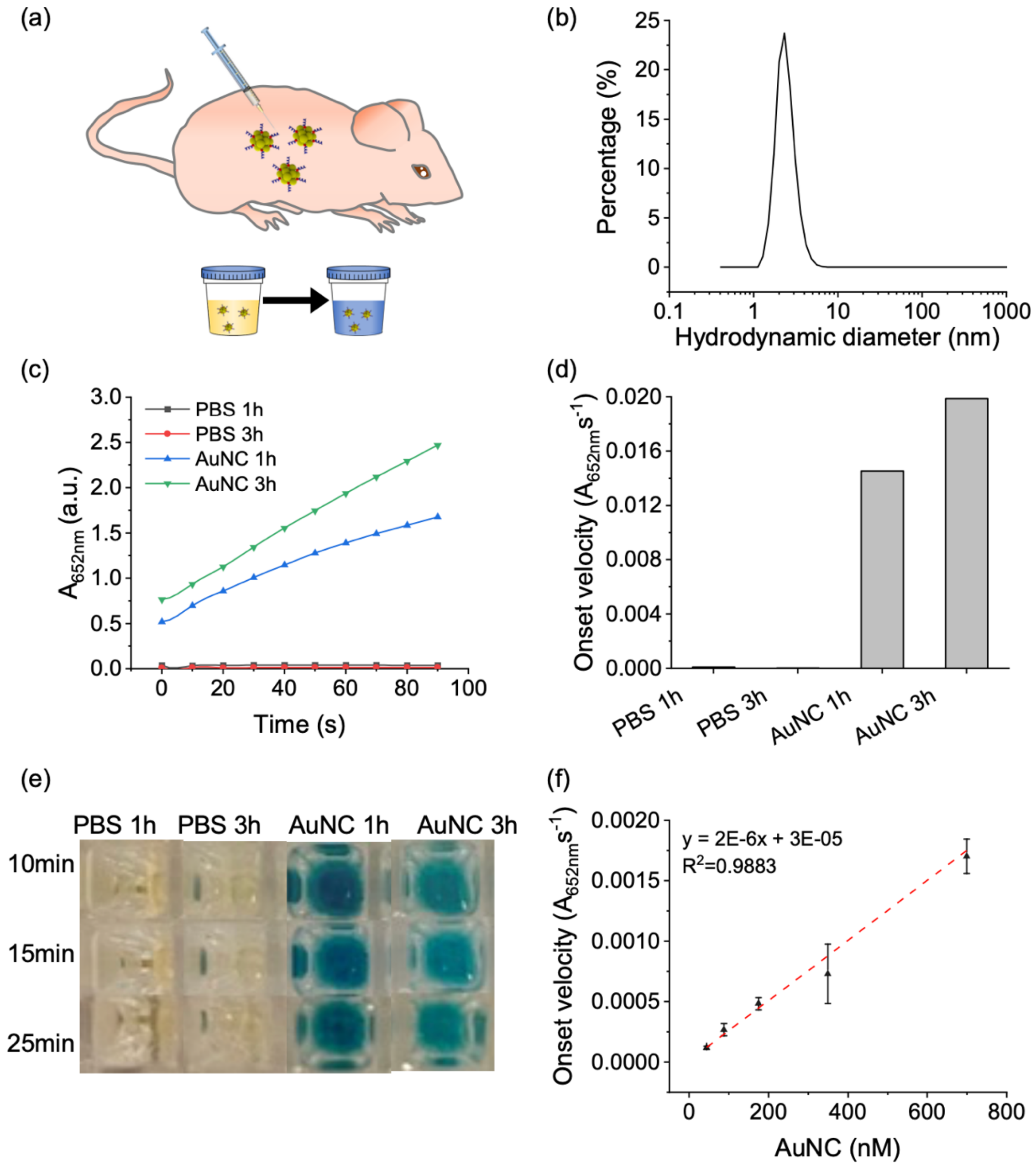

Supplementary Figure 24. Renal clearance study of AuNCs in mice. (a) Schematic of i.p. administration of AuNCs in mice and TMB assay on collected urine. (b) DLS number distribution of AuNC batch used (the curve is mean of n = 3 measurements). (c) Absorbance over time of TMB assay performed on collected urine from PBS control and AuNC-administrated mouse, respectively (n = 1 technical repeat). (d) Onset velocity calculated from (c) and corrected for difference in urine volumes used in assay (10 μL each diluted urine was mixed with 20 μL of 1-Step TMB and 20 μL of 5 M H_2O_2, n = 1 technical repeats). (e) Photograph for (c, d). (f) Calibration curve for AuNCs in synthetic urine used for clearance percentage calculation (mean values ± standard error, n = 3 technical repeats).

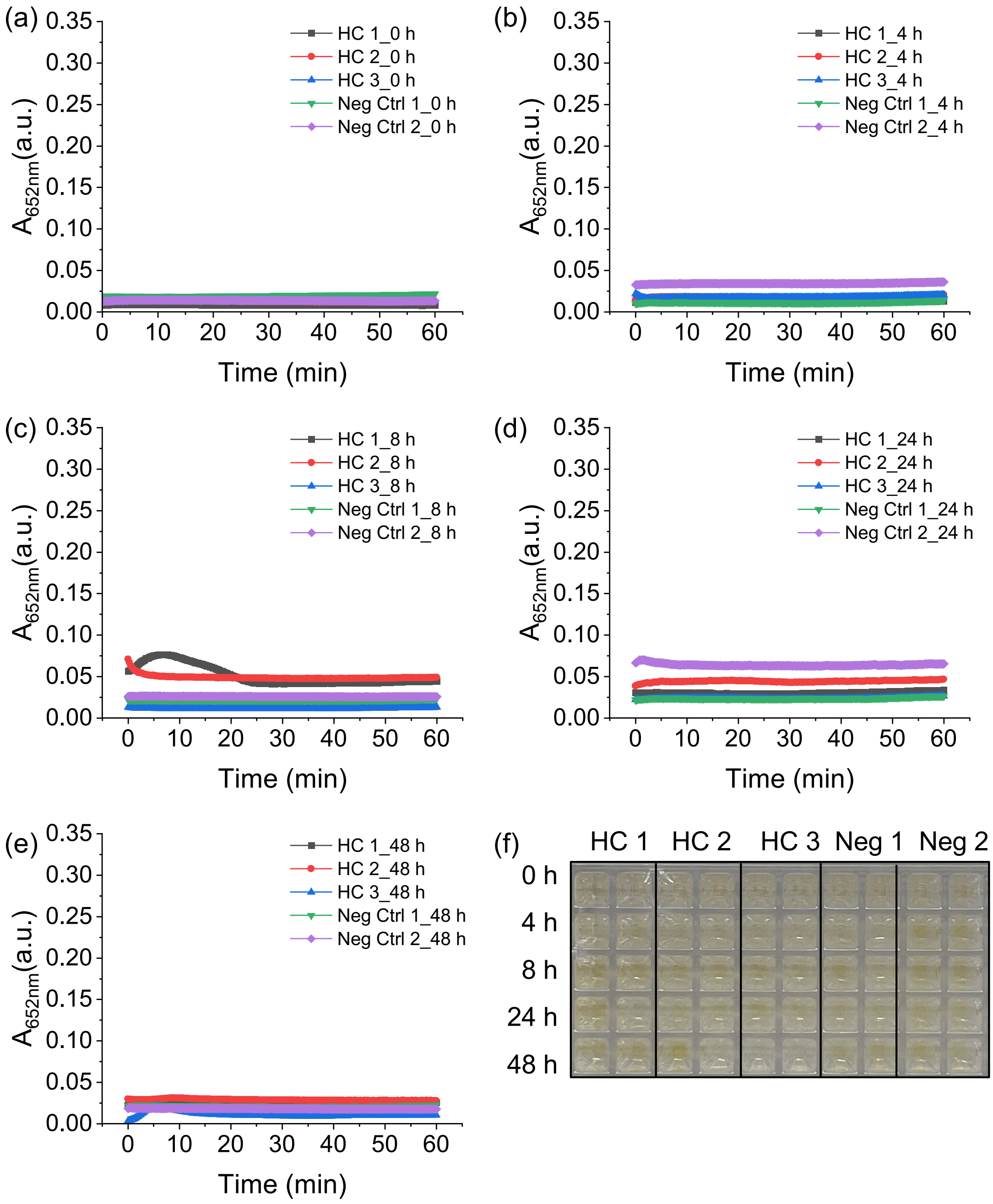

Supplementary Figure 25. ***In vivo*** **stability of liposomal sensor post i.p. administration with HA gel in mice after 0 to 48 hours.** Absorbance of TMB assay performed on collected urine post i.p. administration of HA gels with incorporated liposomal sensor into 3 healthy control (HC) mice and PBS administration into 2 mice as negative control (Neg Ctrl). Urine collected after 0 hour (a), 4 hours (b), 8 hours (c), 24 hours (d), and 48 hours (e). (f) Photographs of TMB assay results from (a-e) showing no blue colour change. There were some artifacts, observed as humps (c-e), which were likely associated with the presence of bubbles in the reaction solution after reagent addition. This does not correspond to a TMB signal, as no accompanying blue

colour change was observed in (f) (for healthy control mice (HC), N = 3 biological replicates, n = 2 technical repeats; for negative control mice (Neg Ctrl), N = 2 biological replicates, n = 2 technical repeats).

Supplementary Movie 1. Opening and closing of a triangular active site on $Au_{25}(SG)_{18}$, as observed in a representative all-atom molecular dynamics simulation. The internal gold–sulphur nanocluster surface features eight equivalent active site pockets. The movie illustrates how the rearrangement of glutathione peptide-ligands influences the solvent-accessible surface area of an individual pocket (shown below atomistic structure). Atoms are displayed in a space-filling representation and coloured by element: oxygen (red), carbon (grey), nitrogen (blue), hydrogen (white), gold (orange), and sulfur (yellow). Water is omitted for clarity.

References

1. Peng, C. *et al.* Targeting orthotopic gliomas with renal-clearable luminescent gold nanoparticles. *Nano Res* **10**, 1366–1376 (2017).

2. Loynachan, C. N. *et al.* Renal clearable catalytic gold nanoclusters for in vivo disease monitoring. *Nat Nanotechnol* **14**, 883–890 (2019).

3. Franco-Ulloa, S., Riccardi, L., Rimembrana, F., Pini, M. & De Vivo, M. NanoModeler: A Webserver for Molecular Simulations and Engineering of Nanoparticles. *J Chem Theory Comput* **15**, 2022–2032 (2019).

4. Brancolini, G., Toroz, D. & Corni, S. Can small hydrophobic gold nanoparticles inhibit β2- microglobulin fibrillation? *Nanoscale* **6**, 7903–7911 (2014).
