# Supplementary material for: Non-invasive in vivo sensing of bacterial implant infection using catalytically-optimised gold nanocluster-loaded liposomes for urinary readout
Source: Nat Commun. 2024 Nov 28;15:10321. doi: 10.1038/s41467-024-53537-2 (PMC11605077; doi:10.1038/s41467-024-53537-2)
Supplement: Supplementary file 3 — Description of Additional Supplementary Files [file 41467_2024_53537_MOESM3_ESM.pdf]

Description of Additional Supplementary Files

Supplementary Movie 1: Opening and closing of a triangular active site on $Au_{25}(SG)_{18}$, as observed in a representative all-atom molecular dynamics simulation. The internal gold–sulphur nanocluster surface features eight equivalent active site pockets. The movie illustrates how the rearrangement of glutathione peptide-ligands influences the solvent-accessible surface area of an individual pocket (shown below atomistic structure). Atoms are displayed in a space-filling representation and coloured by element: oxygen (red), carbon (grey), nitrogen (blue), hydrogen (white), gold (orange), and sulfur (yellow). Water is omitted for clarity.
