# Supplementary material for: Non-invasive in vivo sensing of bacterial implant infection using catalytically-optimised gold nanocluster-loaded liposomes for urinary readout
Source: Nat Commun. 2024 Nov 28;15:10321. doi: 10.1038/s41467-024-53537-2 (PMC11605077; doi:10.1038/s41467-024-53537-2)
Supplement: Supplementary file 5 — Reporting Summary [file 41467_2024_53537_MOESM5_ESM.pdf]

nature portfolio

Corresponding author(s): Adrian Najer, Irene Yarovsky, Molly M. Stevens

Last updated by author(s): Sep 7, 2024

Reporting Summary

Nature Portfolio wishes to improve the reproducibility of the work that we publish. This form provides structure for consistency and transparency in reporting. For further information on Nature Portfolio policies, see our Editorial Policies and the Editorial Policy Checklist.

Statistics

For all statistical analyses, confirm that the following items are present in the figure legend, table legend, main text, or Methods section.

n/a	Confirmed	
☐	☒	The exact sample size (n) for each experimental group/condition, given as a discrete number and unit of measurement
☐	☒	A statement on whether measurements were taken from distinct samples or whether the same sample was measured repeatedly
☐	☒	The statistical test(s) used AND whether they are one- or two-sided<br>*Only common tests should be described solely by name; describe more complex techniques in the Methods section.*
☒	☐	A description of all covariates tested
☐	☒	A description of any assumptions or corrections, such as tests of normality and adjustment for multiple comparisons
☐	☒	A full description of the statistical parameters including central tendency (e.g. means) or other basic estimates (e.g. regression coefficient) AND variation (e.g. standard deviation) or associated estimates of uncertainty (e.g. confidence intervals)
☐	☒	For null hypothesis testing, the test statistic (e.g. F, t, r) with confidence intervals, effect sizes, degrees of freedom and P value noted<br>*Give P values as exact values whenever suitable.*
☒	☐	For Bayesian analysis, information on the choice of priors and Markov chain Monte Carlo settings
☒	☐	For hierarchical and complex designs, identification of the appropriate level for tests and full reporting of outcomes
☒	☐	Estimates of effect sizes (e.g. Cohen's d, Pearson's r), indicating how they were calculated

Our web collection on statistics for biologists contains articles on many of the points above.

Software and code

Policy information about availability of computer code

Data collection	SoftMax Pro (Version 5.4) was employed to collect data on SpectraMax M5 multimode microplate reader (Molecular Devices, Ltd.). Malvern Zetasizer software (7.13) was used for DLS/Zeta measurements. ZEN Software (Carl Zeiss, Jena, Germany) was used to record FCS/FCCS data. iTEVA iCAP Software (Version 2.2.0.51) was used for ICP-MS measurement. MALDI Solutions Data Acquisition Software was used to collect date on Shimadzu MALDI-8030 mass spectrometer. NanoSight NTA software (Version 3.2) was employed to collect data on NanoSight NS300.
Data analysis	ZEN Software (Carl Zeiss, Jena, Germany) and PyCorrFit (Version 1.1.6) were used for FCS analysis (details in Methods section). Custom MATLAB scripts were used for SPARTA analysis. The GROMACS 2022 software package was used for All-atom MD simulations in explicit aqueous solvent. Power Point (Version 16) was used for the process of TEM/Cryo-TEM images. Microsoft Excel (Version 16) and OriginPro 2023 (Version 10.0.0.154) were used for all data analysis and statistics.

For manuscripts utilizing custom algorithms or software that are central to the research but not yet described in published literature, software must be made available to editors and reviewers. We strongly encourage code deposition in a community repository (e.g. GitHub). See the Nature Portfolio guidelines for submitting code & software for further information.

Data

Policy information about availability of data

All manuscripts must include a data availability statement. This statement should provide the following information, where applicable:

- Accession codes, unique identifiers, or web links for publicly available datasets
- A description of any restrictions on data availability
- For clinical datasets or third party data, please ensure that the statement adheres to our policy

Experimental raw data and molecular simulation data are available upon request from the corresponding authors.

Field-specific reporting

Please select the one below that is the best fit for your research. If you are not sure, read the appropriate sections before making your selection.

☒ Life sciences ☐ Behavioural & social sciences ☐ Ecological, evolutionary & environmental sciences

For a reference copy of the document with all sections, see nature.com/documents/nr-reporting-summary-flat.pdf

Life sciences study design

All studies must disclose on these points even when the disclosure is negative.

Sample size	Sample size is given in the figure legends for each experiment. Sample-size calculations were not performed. Sample size was minimised taking into consideration the variability of bacteraemia between animals.
Data exclusions	No data was excluded for in vitro experiments. No animals were excluded from mouse trials.
Replication	Numbers of replicates are given in figure legends and text.
Randomization	Mice were randomised before injections.
Blinding	The expert performing injections in mice was blinded to the treatment groups.

Reporting for specific materials, systems and methods

We require information from authors about some types of materials, experimental systems and methods used in many studies. Here, indicate whether each material, system or method listed is relevant to your study. If you are not sure if a list item applies to your research, read the appropriate section before selecting a response.

Materials & experimental systems

n/a	Involved in the study	
☒	☐	Antibodies
☐	☒	Eukaryotic cell lines
☒	☐	Palaeontology and archaeology
☐	☒	Animals and other organisms
☒	☐	Human research participants
☒	☐	Clinical data
☒	☐	Dual use research of concern

Methods

n/a	Involved in the study	
☒	☐	ChIP-seq
☒	☐	Flow cytometry
☒	☐	MRI-based neuroimaging

Eukaryotic cell lines

Policy information about cell lines

Cell line source(s)	RAW 264.7 cells (ATCC TIB-71)
Authentication	No further authentication was performed, since these are commercially available cell lines.
Mycoplasma contamination	All cell lines tested negative for mycoplasma contamination.
Commonly misidentified lines (See ICLAC register)	No commonly misidentified lines were used in the study.

Animals and other organisms

Policy information about studies involving animals; ARRIVE guidelines recommended for reporting animal research

Laboratory animals	Wild-type C57BL/6 mice were used in this study.
Wild animals	This study did not use wild animals.
Field-collected samples	This study did not involve field-collected samples.
Ethics oversight	Mouse experiments were conducted according to the Animals (Scientific Procedures) Act 1986, following ethical review by Imperial College London Animal Welfare and Ethical Review Body (PF93C158E and PP5168779), and with approval from the UK Home Office authority.

Note that full information on the approval of the study protocol must also be provided in the manuscript.
